# Drug company payments to General Practices in England: Cross-sectional and social network analysis

**DOI:** 10.1371/journal.pone.0261077

**Published:** 2021-12-07

**Authors:** Eszter Saghy, Shai Mulinari, Piotr Ozieranski

**Affiliations:** 1 Faculty of Pharmacy, Division of Pharmacoeconomics, University of Pecs, Pecs, Hungary; 2 Department of Sociology, Lund University, Lund, Sweden; 3 Department of Social and Policy Sciences, University of Bath, Bath, United Kingdom; York University, CANADA

## Abstract

Although there has been extensive research on pharmaceutical industry payments to healthcare professionals, healthcare organisations with key roles in health systems have received little attention. We seek to contribute to addressing this gap in research by examining drug company payments to General Practices in England in 2015. We combine a publicly available payments database managed by the pharmaceutical industry with datasets covering key practice characteristics. We find that practices were an important target of company payments, receiving £2,726,018, equivalent to 6.5% of the value of payments to all healthcare organisations in England. Payments to practices were highly concentrated and specific companies were also highly dominant. The top 10 donors and the top 10 recipients amassed 87.9% and 13.6% of the value of payments, respectively. Practices with more patients, a greater proportion of elderly patients, and those in more affluent areas received significantly more payments on average. However, the patterns of payments were similar across England’s regions. We also found that company networks–established by making payments to the same practices–were largely dominated by a single company, which was also by far the biggest donor. Greater policy attention is required to the risk of financial dependency and conflicts of interests that might arise from payments to practices and to organisational conflicts of interests more broadly. Our research also demonstrates that the comprehensiveness and quality of payment data disclosed via industry self-regulatory arrangements needs improvement. More interconnectivity between payment data and other datasets is needed to capture company marketing strategies systematically.

## Introduction

Drug company payments to the healthcare sector can create conflicts of interest biasing clinical practice [1], research [2,3], and policymaking [4]. A key global trend towards addressing this risk involves payment disclosure via either public regulation (e.g., the US Open Payments or French Transparence Sante databases [5]) or industry self-regulation (e.g., most European countries, including the UK [6], Japan [7], and Australia [5]).

Research on payment disclosures has centered on *individual healthcare professionals* [8–11], with increasing evidence from the US of even small payments influencing drug prescription [12–14] and healthcare cost [14,15]. However, *healthcare organisations* (HCOs), including service providers, regulators or medical societies, have received less attention, even though they shape healthcare delivery via resource allocation, regulatory decisions, recommendations and guidelines [16,17]. The limited interest in payments to HCOs in the US [18] seems to reflect the fact that the Sunshine Act only covers payments to hospitals. However, the definition of organisational-level recipients adopted in European countries with self-regulation is conisderably broader, therefore allowing for capturing the unique compositions of HCOs in national healthcare systems [21]. Additionally, pharmaceutical companies and trade groups typically do not interpret payments to HCOs as falling under European data privacy laws, which prevents these recipeints from refusing to have their payments disclosed [17]. This contrasts with payments to healthcare professionals, interpreted by the industry as “personal data”, and therefore characterised by pervasive non-disclosure, precluding comprehensive analysis [11]. For example, in 2015 in the UK, only around 50% of the disclosed payments had any information about the individual recipients [10], and this had increased only to about 60% by 2019 [11].

Despite the relatively greater data availability, the UK is the only European country with patterns of payments to HCOs described at the national level [19] and in relation to organisations commissioning (or procuring) healthcare services for patients [20,21] as well as secondary-care providers [22]. Building on this research, we examine payments to General Practice (GP) surgeries (henceforth, practices), excluding specialist practices providing services related to specific fields of medicine [23]. As healthcare is organised differently across the UK [24], we examine England as its largest part. We focus on practices given their vital role in healthcare delivery in England, with over 60 million patients being registered at practices [25] and over 300 million appointments annually, compared to 23 million accident and emergency service hospital visits [26]. Further, over half of the total National Health Service (NHS) pharmaceutical spending involved prescriptions issued by practices [26].

We anticipate that practices will be a key target of company payments. Consistent with patterns of payments to HCOs in the UK [19] and the US [18], we also expect a few companies and recipients to concentrate most payments. Furthermore, following US research emphasising the importance of relatively small payments in influencing physicians [18,27,28], we predict that most companies will make many relatively small payments rather than a few large payments.

We also consider key practice characteristics–location, size, and some features of the patient population–as potentially affecting company choices about who receives payments. We hypothesise that the *proportion* of practices receiving payments is roughly equal across the regions of England. However, we expect to see differences in the *amount* of payments between practices in different parts of England reflecting previously demonstrated regional variation in prescribing patterns [29]. We anticipate more payments to practices with a higher number of registered patients compared to those with a smaller clientele, given the predicted greater “return on investment” for companies. Specifically, we expect that practices with higher shares of patients over the age of 65 will receive more payments compared to those with fewer elderly patients, due to, for example, the greater tendency for polypharmacy in elderly populations [30]. Moreover, we expect practices in more deprived locations to obtain more payments compared to those in more affluent areas [31] as, for example, studies in Northern Ireland [32] and Scotland [33] found greater numbers of prescriptions per patient in the most deprived areas compared with the least deprived ones, making them potentially more attractive as payment recipients.

Given the well-documented “relational” nature of the pharmaceutical industry, including its attempts to develop ties to, and visibility among, actors seen as vital for driving product uptake and profitability [34–37], we use social network analysis (SNA) to explore social structures involved in making payments to practices. Drawing on emerging applications of SNA to study pharmaceutical industry payments and marketing [38], we anticipate that connections established by making payments to practices are not accidental. For example, data analytics companies have offered SNA insights to map Key Opinion Leaders (KOLs) in the medical field in the US [38–41], and it is likely that similar services are also used in European countries [40,41]. As data disclosed within industry self-regulation has no information on products related to payments [6,19], SNA cannot trace product competition among companies. Instead, we examine i) which companies are interested in making payments to the same practices, ii) which companies are dominating the payment networks (if any), and iii) the differential density of connections within such networks. In so doing, we consider two types of networks. We interpret networks based on the *value* of payments as indicating the “importance” of a practice for a drug company, while networks involving the *number* of payments as pointing to the intensity of interactions with the practice. More frequent payments may, for example, enhance a company’s visibility, which could be an important goal of marketing efforts [42].

We had two specific objectives. We sought to analyse, first, the distribution of and factors associated with payments across drug companies and practices in England; and, second, the structure of connections between drug companies established by making payments to the same practices.

## Methods

### Study design

Our study combines cross-sectional and SNA analysis of drug company payments to practices in England. We combined Disclosure UK [43]–an annually published dataset including, among others, non-research payments to named HCOs, disclosed by companies following the Code of Practice of the Association of the British Pharmaceutical Industry (ABPI) [19]–with separately sourced information on practice characteristics. We analysed the distribution of payments across practices and companies and assessed the associations with selected practice characteristics. We then mapped the structure of connections between companies and their shared practices using SNA. Given the exploratory nature of our research, which involved combining datasets which had not been previously used to analyse drug company payments, our study did not follow an a priori protocol.

### Data sources and extraction

We extracted data on company payments to practices from the 2015 edition of Disclosure UK as this is the only one for which previous research categorised payment recipients, which enabled isolating payments to practices [19]. The relationship between practices and the previously examined larger category of public sector primary care providers [19] is explained in S1 Appendix. To prevent any payments to practices from being unnecessarily excluded due to companies potentially misidentifying their ultimate recipients [19], we combined payments practices identified in either “Institution name” or “Institution location” columns of the dataset (2,945 payments in total, out of which 2,747 were used for the analysis). The section of Disclosure UK we analysed (Online supplement 1) can be matched with Disclosure UK version 20160630. To allow accurate comparison of payment values between companies we adjusted them for VAT using information from company "methodological notes”, as described elsewhere [19].

We used the GP Friends and Family Test (FFT) dataset [44] to assign unique codes to practices identified in Disclosure UK. The practice names were matched with the practice codes based on comparing practice names and addresses from the two datasets. For each practice with a unique code we obtained the number of registered patients using the Patients Registered at a GP Practice 2015 NHS dataset [25]. We also used this dataset to calculate the share of patients over 65. In addition, we obtained multiple deprivation index (MDI) decile scores for the postcode of each practice from the website of the Ministry of Housing, Communities and Local Government [45]. The MDI is an aggregated score of 37 indicators providing information on income; employment; health and disability; education, skills and training; crime; barriers to housing and services and living environment [46]. We divided practices into 4 quartiles based on their MDI score.

The categorisation and cleaning of drug company payment data is described elsewhere [42]. The extraction of data from the datasets with practice characteristics is described in the protocol available in S2 Appendix.

### Analysis

#### Statistical analysis

We used R [47] version 1.4.1717 to analyse the distribution of payments descriptively and to assess differences across selected practice characteristics. As the distribution was heavily skewed, we examined medians and interquartile ranges. Significance of the difference in the value of payments between different groups was assessed using the Wilcoxon nonparametric statistical test. The reference groups are London in regional comparison; Lowest number of patients (1^*st*^ quartile) in practice size comparison; Lowest share of elderly patients (1^*st*^ quartile) in elderly patient population comparison, and Most deprived (1^*st*^ quartile) in MDI comparison. The threshold for significance (alpha) was set to 0.05.

#### Social network analysis

We first created company by practice matrices in MS Excel, then converting them into company by company matrices to allow for examining connections between companies established by “shared” practices, i.e., practices to which any pair of companies made payments. We report findings calculated based on “valued” matrices, with the number of shared practices shown at the intersect of companies. We created separate matrices for different thresholds of the number and value of payments involved in establishing connections between companies; quartiles of the overall number and value of payments per company; and practice characteristics (i.e., quartiles of the total number of patients; quartiles of the share of patients over 65; and quartiles of the MDI of the postcodes in which the practices were based) (Online supplement 2).

We analysed the matrices in UCINET version 6.689 [48], visualising them in Gephi version 0.9.2. [49]. We calculated each company’s *centrality*, which is the number of ties a company has, i.e. the number of connections to other companies established by making payments to the same practices [50]. We also calculated network *centralisation*, showing, on a scale from 0 to 1, the extent to which a network is dominated by one company [50]. Centralisation score is measured as the ratio of the actual sum of centrality score differences and all possible sum of centrality score differences [51]. Finally, we calculated network *density*–the strength of existing ties between actors as a share of all possible ties. In our valued networks, density is calculated by dividing the sum of shared practices between all companies in a network by the total of all possible connections [52]. We report findings relating to networks established based on the *value* of payments made by drug companies but throughout the results we also signpost to web appendices with additional findings relating to networks considering the *number* of payments. Overall, from the SNA perspective, we expect to be able to detect companies dominating the field of payments to practices and patterns of lower and higher number of shared practices based on different payment sizes and practice characteristics mentioned above.

## Results

### Descriptive analysis of the distribution of payments to practices

In total, 37 drug companies made 2,945 payments, worth £ 2,726,017.77 to 1,790 practices. In 2015, these companies represented 37.0% of those reporting payments to HCOs in England. Consistent with our expectations regarding the importance of practices as a target of industry payments, payments to practices constituted 6.5% of the value of all payments made to HCOs in England (S3 Appendix) and practices ranked 5^th^ of all HCOs receiving the highest amount of payments after universities, NHS foundation trusts, NHS trusts, and multi-professional organisations.

We excluded from further analysis 198 payments (6.72%), worth £166,351.74 (6.1%) made to 147 (8.21%) practices as we could not link them to practice codes. These practices were randomly distributed across England (S4 Appendix), with the median payment values similar to those in the rest of the dataset. Our final sample, therefore, comprised 2,747 payments worth, £2,559,666.03, made by 34 companies to 1,643 practices. These payments were for donations and grants (76.36%), contributions to costs of events (22.51%), and fees for service and consultancy (1.14%) (S5 Appendix).

As expected, payments were highly concentrated (Table 1). Although three-quarters of practices received no more than two, the top practice received as many as 132. Most companies were “small donors”, with three-quarters making no more than 81 payments, but the maximum number was almost a thousand (i.e., Bayer). The value of payments was similarly concentrated. Although three-quarters of practices received no more than £1,5k, the top one accumulated almost ten times more. Likewise, while three-quarters of companies made payments worth no more than £100k, those made by the top donor were worth more than 7.5 times as much. Three-quarters of companies made payments to no more than 56 practices, but the top donor, Bayer, remarkably, made its 998 payments to 778 practices (2.47% of the value of Bayer’s payments were contributions to costs of events, 96.19% were donations and grants, and 1.34% were fees for service and concultancy). A majority of practices only received payments from one company, while the the top recipient received payments in total from 18 companies.

**Table 1:** Summary of drug company payments to general practices.

*Level of analysis*	*Minimum*	*Median [IQR]*	*Maximum*
*Single payment value (£)*	8.00	320.00 [170.00–869.00]	49,420.80
*Value of payments per general practice (£)*	9.59	576.00 [217.25–1,520.75]	148,395.20
*Number of payments per general practice*	1.00	1.00 [1.00–2.00]	132.00
*Value of payments per company (£)*	80.00	9,036.00 [1,003.00–97,377.00]	765,987.77
*Number of payments per company*	1.00	14.50 [3.25–80.75]	998.00
*Number of practices per company*	1.00	8.50 [3.00–56.00]	778.00
*Number of companies per practice*	1.00	1.00 [1.00–1.00]	18.00

Notes: This table is based on drug company payments reported in Disclosure UK (2015, version 20160630).

Table 2 further evidences the concentration of payments, with those made by the top ten donors constituting, respectively, 93.64% and 83.69% of the total number and value of payments made to practices. Of the 10 companies, Bayer was dominant in the number and value of payments, as well as the number of practices to which payments were made. A similar table including the top 10 recipients is presented in S6 Appendix.

**Table 2:** Payments made by the top 10 drug company donors to general practices.

*Company*	*Total value of payments (£)*	*Total number of payments*	*Number of practices paid*	*Median value of single payments (£) [IQR]*
*Bayer*	765,987.77	998	773	434.50 [217.20–869.00]
*Pfizer*	360,556.90	140	105	1,412.10 [236.00–3,907.00]
*Eli Lilly*	271,139.00	260	185	200.00 [168.00–3,353.00]
*Sanofi Aventis*	269,965.82	149	126	1,000.00 [240.00–2,400.00]
*AstraZeneca*	153,865.25	85	16	250.00 [150.00–550.00]
*Boehringer Ingelheim*	145,070.58	213	146	392.00 [177.60–640.00]
*Merck Sharp & Dohme*	124,062.80	63	50	800.00 [195.80–4,400.00]
*Takeda*	112,428.80	94	58	240.00 [180.00–921.40]
*Napp*	97,743.39	235	212	38.49.00 [28.90–111.73]
*Servier*	96,162.40	62	61	1,567.00 [576.00–1,567.00]

Notes: This table is based on Disclosure UK (2015, version 20160630).

The evidence of companies’ preference for small payments was mixed. Most payments were indeed relatively small, with three-quarters being no more than £869.0 (Table 1). However, important differences existed among the biggest donors (Table 2). Despite the varying overall size of payments per company, the comparison of median payment values shows that companies such as Pfizer, Merck Sharp & Dohme, and Servier made fewer but more substantive payments, while Bayer, AstraZeneca and Napp made a larger number of smaller payments. However, the comparison of the median values at the payment and practice levels suggests there were two groups of companies prioritising small payments, with one concentrating on a smaller number of practices, while the other dispersing its payments across a larger number of practices. For example, while AstraZeneca made 85 payments to 16 practices, Servier made 62 payments to 61 practices. This reflects the overall payment distribution (Table 1), with three-quarters of practices receiving no more than two payments.

The relationships between payment patterns and practice characteristics were broadly consistent with our expectations (Table 3). In most regions of England, the shares of practices receiving payments ranged between a fifth and a quarter of all practices. The only two regions with markedly lower shares were London (8.88%) and North East England (14.66%). Nevertheless, the number of practices receiving payments varied considerably between the regions, with only 107 located in North East England and 267 in North West England. The median payment values also displayed regional differences, with practices in North East England having the median value almost twice as high as those in London and South East England (London vs. North East England; p <0.001). Moreover, the median value of payments per practice increased together with the practice size and the proportion of patients over 65 (all other quartiles are significantly different from the first quartile). However, unexpectedly, practices in the most deprived areas (1^st^ quartile based on MDI) received significantly smaller payments than other practices.

**Table 3:** Breakdown of drug company payments according to general practice characteristics.

*Classification*	*Group*	*Median value of payments (£) [IQR]*	*P-value*	*Number of general practices receiving payments (% out of total practices in the region)*
	London	434.50 [217.25–2,600.00]	Ref	140 (8.88%)
*Regional breakdown*	East Midlands	434.50 [217.25–869.00]	0.756	147 (19.57%)
	East of England	600.00 [208.63–1,086.25]	0.994	136 (19.26%)
	North East England	869.00 [434.50–2,909.12]	<0.001	107 (14.66%)
	North West England	665.88 [217.25–3,168.95]	<0.001	261 (19.30%)
	South East England	434.50 [182.04–910.70]	0.086	249 (26.57%)
	South West England	651.75 [320.00–1,104.00]	0.221	168 (23.90%)
	West Midlands	461.42 [164.00–1,344.00]	0.826	220 (23.63%)
	Yorkshire and the Humber	460.00 [217.23–2,422.12]	0.243	215 (no data)
	Lowest number of patients (1^st^ quartile)	217.25 [82.00–245.59]	Ref	
*Breakdown based on number of registered patients*	Lower number of patients (2^nd^ quartile)	434.50 [200.00–651.75]	<0.001	
	Higher number of patients (3^rd^ quartile)	869.00 [486.50–2,175.00]	<0.001	
	Highest number of patients (4^th^ quartile)	2087.20 [1,012.00–4,400.00]	<0.001	
	Lowest share of elderly patients (1^st^ quartile)	320.00 [167.50–863.12]	Ref	
*Breakdown based on share of patients over 65 years*	Lower share of elderly patients (2^nd^ quartile)	585.00 [217.25–1,409.56]	<0.001	
	Higher share of elderly patients (3^rd^ quartile)	651.75 [242.50–1,864.25]	<0.001	
	Highest share of elderly patients (4^th^ quartile)	869.00 [434.50–2,283.12]	<0.001	
	Most deprived (1^st^ quartile)	434.50 [200.00–1,157.68]	Ref	
*Breakdown based on index of multiple deprivation*	More deprived (2^nd^ quartile)	587.70 [217.25–1,470.70]	0.041	
	Less deprived (3^rd^ quartile)	651.75 [217.25–1,699.80]	0.001	
	Least deprived (4^th^ quartile)	651.75 [325.75–2,056.00]	<0.001	

Notes: The share of the number of practices out of the total were only included for the regional breakdown because data could only be extracted for this variable. We did not find data on the number of practices in Yorkshire and the Humber. These practices are possible counted together with practices in North East England. Significance of the difference in the value of payments between different groups was assessed using Wilcoxon nonparametric statistical test. Reference groups are London, Lowest number of patients (1^st^ quartile), Lowest share of elderly patients (1^st^ quartile), and Most deprived (1^st^ quartile). This table is based on Disclosure UK (2015, version 20160630), the GP Friends and Family Test (FFT) dataset, and the Patients Registered at a GP Practice 2015 NHS dataset.

### Social network analysis of connections between drug companies

**Fig 1:**
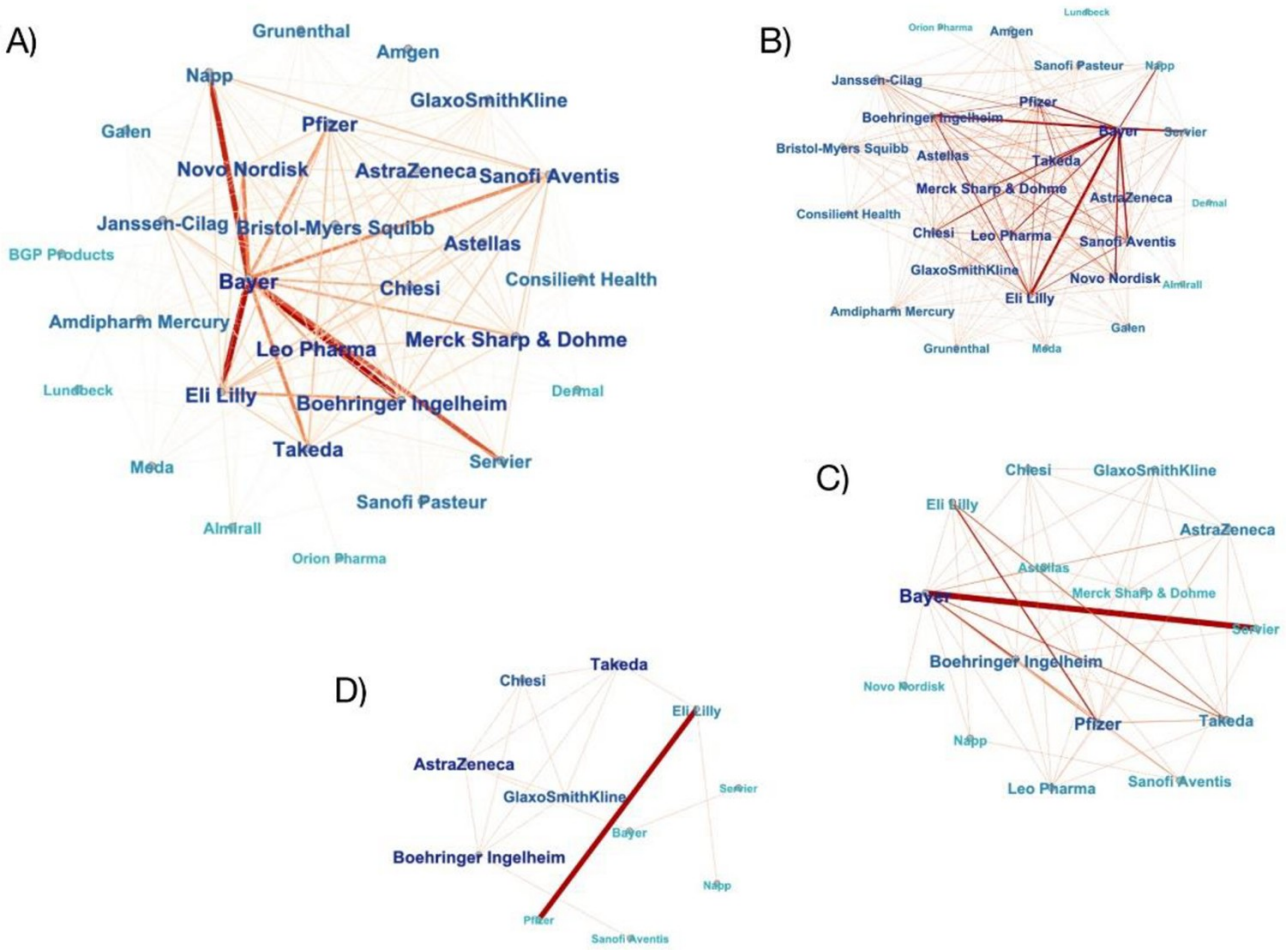
Networks based on the value of payments. Notes: 1A) network of all payments; 1B) network of payment over £100 per practice; 1C) network of payment over £1000 per practice; 1D) network of payment over £2500 per practice. Fig 1A–1D shows the visualisation of networks based on the value of payments, created in Gephi. Node label size and darkness corresponds to the centrality of a company, the strength and darkness of the lines corresponds to the number of shared practices between companies. The networks visibly change as the payment number to a single practice increases.

Fig 1 shows valued networks of connections between companies making payments to the same practices. A connection indicates at least one payment made to the same practice and line thickness and darker colour correspond with a greater number of shared practices. Therefore, companies connected with thicker and darker lines can be interpreted as having a shared interest in a greater number of practices. We demonstrate configurations of companies at different level of the value of payments. In Fig 1A, all companies are shown, while in Fig 1B–1D only companies making individual payments worth at least £100, £1000, and £2,500, respectively, are shown.

As the value of payments increased, the number of companies decreased from 29 (Fig 1A) to 11 (Fig 1D), suggesting that only a few companies engaged with practices using high-value payments. The configurations of companies also changed, indicating similarities and differences in how they engaged with practices with payments above a certain value.

Fig 1A–1D can also be analysed in terms of their density, where higher density of a graph means stronger connections between a greater proportion of companies. The density of the graphs decreases from A to D, as the value of payments increases (see density scores in S7 Appendix). This suggests that there is an overall lower interest in the same practices as expressed by higher value payments, which means that one practice does not usually receive high value payments from multiple companies.

Moving on to specific companies, considering all payments, Bayer had the greatest shared interest in practices with Napp, Eli Lilly and Boehringer Ingelheim (Fig 1A). But when only accounting for payments worth over £1,000, Bayer made the highest number of payments to the same practices with Servier. In addition, among companies making the highest-value payments, over £2,500, those with the greatest shared interest in practices were Eli Lilly and Pfizer (Fig 1D).

This trend corresponds with companies’ centrality scores, which rose with the increasing number of connections with other companies. In Fig 1A–1C, Bayer is the company with the highest centrality score, while in Fig 1D Eli Lilly is the most central one of the network (see centrality scores in S7 Appendix).

The centralisation level of a graph indicates the extent to which one company dominates a network by being connected to a high number of companies, while other companies have less connections. From the four networks, Fig 1A is the most centralised, with Bayer dominating the network. This means that on many occasions when a company makes payments to a practice, Bayer also makes a payment there. Similarly to density, centralisation decreases as the value of payments increases (see centralisation scores in S7 Appendix). Further graphs of networks based on the number of payments can be found in S8 Appendix.

In S9 Appendix, we present additional results for valued drug company networks associated with making payments to practices with different characteristics. Interesting differences can be observed in terms of centralisation (the extent to which one company dominates a network) and centrality (the number of connections a company has), which we are reporting here, while differences between networks in density are not substantial. Regarding the region in which the practices were located, the highest centralisation was observed in South West of England, while the lowest–in South East England. Across the nine regions of England, Bayer had the highest centrality scores, with only Eli Lilly matching it in London and South East England. In company networks established based on making payments to practices of different sizes, a trend existed of increasing network centralisation as the number of patients increased, with the highest centralisation score in the third quartile of practice size. Bayer was, again, the most central company in all four (quartile 1 –quartile 4) networks showing payments made to practices with different patient numbers. In the networks involving payments made to practices based on the proportion of patients over the age of 65, the centralisation score does not change substantially with the increase in the proportion of elderly patients. Bayer also remained the company with the highest centrality score in all quartiles. A similar trend exists in networks with the MDI index (1^st^ quartile being the most deprived) of the location of the practice. Bayer was, yet again, equally dominant in the most and least deprived areas.

## Discussion

As far as we know, this is the first study examining drug company payments to the primary care sector. We find that general practices were a major target of industry payments in England, placing them in the top five and two of organisational recipients based on the value and number of payments, respectively (S3 Appendix). While the value of payments received by general practicioners is unknown given the big gaps in individual-level payment data, they could exceed considerably the organisational-level payments to practices [10]. Notably, the payments made to practices in England (£2.7m) were almost twenty times lower than those made to individual healthcare professionals in UK in 2015 (£50.9m) [10]. Overall, our findings suggest that more attention is needed to drug company payments to organisations and to organisational conflicts of interest [53–55].

Turning to the modes of financial engagement with practices, the high value of “grants and donations” (almost 65%) suggests that companies often provided them with “medical and educational goods and services”, which may bear company names but not product names [56]. Contrastingly, the low value of consultancy payments (less than 2%) suggests limited scope of practices offering, on behalf of their employees, services such as “market research” (defined broadly as “the collection and analysis of information” on medicines) or “chairing and speaking at meetings, assistance with training and participation in advisory boards” [56]. “Contributions to cost of events”, accounting for around a third of payments, covers events, such as conferences, organised by practices or third parties on their behalf.

Payments to practices were highly concentrated, just like in the UK overall [19]. From the industry side, more than a third (37%) of all companies making payments to HCOs in England reported having made payments to practices. Only a few companies were big donors, with the payment landscape largely dominated by one company, Bayer, which, incidentally, was also identified as the second largest source of payments to healthcare professionals in the UK in 2015 [10]. Bayer was dominanant across all regions of England, practice sizes, and patient population profiles. The SNA provided further evidence of concentration of payments among companies.

We also saw concentration of payments among practices, with many receiving only small or occasional payments, yet with a narrow subset being heavily exposed to industry funding. Although the conference or education budgets of the top recipients are unknown, the volume of reported payments suggests that the industry–or, indeed, specific companies–were a major source of such support. This is important as research on drug company funding within the healthcare sector [5], including patient organisations [57], highlights risks associated with dependency on industry funding, especially coming from a few donors.

Not only have we found significant regional differences in payment values received by practices across England, but we have also revealed that practices with the lowest number of patients, the lowest share of elderly patients, and those in the most deprived areas receive significantly lower amount of payments. Why practices in most most deprived areas recive less industry funding, and the consequence of this for general practicioners and their patients, should be investigated further.

We identified some evidence of the high-frequency but low-value payment strategy, which has been highlighted as potentially instrumental in generating networks of obligation with US healthcare professionals [58–62]. Here, we unearthed some divergence within this strategy, with some companies making many small payments to different practices, while others concentrating their small payments on fewer practices.

While these differences may indicate contrasting marketing strategies, our interpretation is constrained by the absence of information on products related to payments. Therefore, unlike with meals and small gifts reported in relation to US physicians [6,19,28,59], we do not know the significance of “small” payments, for example, for establishing extended reciprocity at the organisational level. Investigation of payment strategies would be *even less* possible in other European countries with self-regulation of payment disclosure. This is because the ABPI is the only European pharmaceutical industry trade group mandating its member companies not to aggregate payments to HCOs annually per recipient, which allows comparing payments of different sizes. While we are not aware of any detailed guidance from the ABPI associated with this requirement, a review of cases from the UK drug industry self-regulatory authority, the PMCPA, has not identified any relevant compliants. Therefore, it is unlikely that companies had difficulties in interpreting how payments to HCOs should be itemised.

While the key issue of company marketing cannot be addressed directly in the European self-regulatory context, previous research on European self-regulatory systems has captured companies’ marketing *indirectly* by considering the nature and frequency of investigations into unethical marketing for specific products, highlighting heavy marketing of drugs prescribed in general practice—antidepressants in the late 1990s [63], followed by anti-diabetics and urologics (mainly erectile dysfunction drugs) in the next decade [64]. Similarly, we note that between 2012–2018, Bayer was sanctioned by the PMCPA [65,66] on no less than 12 occasions for unethical marketing of Xarelto (rivaroxaban), a direct oral anticoagulant (DOAC) often prescribed by general practicioners as stroke prophylaxis in patients with atrial fibrillation [67], suggesting that Bayer’s payments to practices could be associated with this drug. Indeed, DOACs have been identified as heavily marketed products in the UK [68].

Finally, the prominence of drug company payments does not seem to be matched adequately by governance frameworks available to practices. Although NHS England requires NHS trusts and clinical commissioning group employees to record externally sponsored events and urges NHS staff to decline gifts that may affect their professional judgement [69], less clarity exists regarding organisational conflicts of interests, which might be associated with payments analysed in our study.

### Limitations

Our article has some important limitations. While the value of disclosed payments to practices is substantial, it excludes payments for research and development, such as clinical and non-clinical studies, which are not disclosed on a named basis in accordance with self-regulatory rules. Moreover, we did not examine conflict of interest reporting by the practices from our dataset, which might reveal payments underreported by donors or recipients, as indicated by comparison of payments reported separately by drug companies and NHS trusts [22] and clinical commissioning groups [20] in England. Our findings are only part of a bigger picture of payments to primary care organisations. For example, companies also make payments to groups of practices or organisations involved in education of general practitioners (see examples in S3 Appendix). Extensive payments are also made to clinical commissioning groups, which procure primary care services across England [20].

Moreover, the selection of practice characteristics was not theoretically driven and omitted other potentially important ones, such as ratings of quality services. Finally, we did not examine the decision processes behind making payments nor those involved in accepting (or refusing) them. Yet, following a recent study on patient organisations, more qualitative research is needed to explore the different patterns of payments and what they might mean for practices and general practicioners and whether, and, if so, how they can influence treatment decisions [70].

### Policy recommendations

The insufficient levels of payment and conflict of interest transparency indentified by our study are concerning, particularly in relation to practices receiving substantial–in the tens of thousands of pounds–annual payments from individual drug companies. Therefore, Disclosure UK should include payment descriptions–similar to those already provided in the self-regulatory arrangements for payments to patient organisations [65]–to illuminate payments’ intended goals. Similarly, without recipient identifiers data users are unable to establish the level of exposure of any practice to drug company payments [11,19]. Consequently, Disclosure UK should introduce identifiers already used by the NHS (i.e., practice codes), which would also allow for linking payment data to other publicly available datasets. Further, information about products associated with payments is necessary to investigate company marketing strategies, as is the case with the government-run US Open Payments Database [71]. In addition, comparing payments made to different HCOs requires the inclusion of recipient categories to avoid the need for checking the nature of and categorising the recipient of each payment [19]. More broadly, payments to HCOs reported in other European countries with self-regulation [6] should be itemised to allow examinining payments of different sizes.

In the long-run, a separate centralised public reporting system by practices is needed, comprising research and non-research payments from pharmaceutical and medical device companies. There are currently voluntary initiatives to make data about the links between doctors and the pharmaceutical industry publicly available, such as the UK’s whopaysthisdoctor.org. However, a central register allowing patients to see the financial interest of all doctors in particular for medicines or medical devices is also being discussed [72]. The establishment of any central payment registers should be coupled with information campaigns directed at medical professionals, patients and members of the public seeking to develop their understanding of conflicts of interests. These steps seems necessary to achieve behavioural change that to-date has not been triggered by the US Open Payments database, as demonstrated by physicians’ continued acceptance of COIs [73] or patients’ and public’s low engagement with payment data [73,74].

Beyond transparency, potential dependency of practices–or some of their activities–on drug company payments requires policy attention. The ABPI has recently acknowledged this problem by prohibiting companies following its Code of Practice from requiring being the sole funders of HCOs [56]. Nevertheless, building on ABPI’s recommendations regarding payments to patient organisations, companies should disclose the share of their payments in relevant organisational budgets [56].

## Supporting information

S1 Appendix Coding of general practices. (DOCX)

S2 Appendix Data cleaning protocol. (DOCX)

S3 Appendix Total value and number of payments to the top 10 healthcare organisations in England in 2015. (DOCX)

S4 Appendix Distribution of practices across different regions England. (DOCX)

S5 Appendix Breakdown of payments types received by general practices. (DOCX)

S6 Appendix Payments to the top ten general practices. (DOCX)

S7 Appendix Summary of network statistics calculated for valued networks of drug companies. (DOCX)

S8 Appendix Network visualizations for networks based on the number of payments. (DOCX)

S9 Appendix Breakdown of network statistics according to general practice characteristics. (DOCX)

## References

[1] Sharma M., et al., Association between industry payments and prescribing costly medications: an observational study using open payments and medicare part D data. BMC health services research, 2018. 18(1): p. 236–236. doi: 10.1186/s12913-018-3043-8 29609611 PMC5880069

[2] Lundh A., et al., Industry sponsorship and research outcome: systematic review with meta-analysis. Intensive care medicine, 2018. 44(10): p. 1603–1612. doi: 10.1007/s00134-018-5293-7 30132025

[3] Liu J.J., et al., Payments by US pharmaceutical and medical device manufacturers to US medical journal editors: retrospective observational study. BMJ (Clinical research ed.), 2017. 359: p. j4619–j4619. doi: 10.1136/bmj.j4619 29074628 PMC5655612

[4] Moynihan R., et al., Financial ties between leaders of influential US professional medical associations and industry: cross sectional study. BMJ, 2020. 369: p. m1505. doi: 10.1136/bmj.m1505 32461201 PMC7251422

[5] Grundy Q., et al., Decoding disclosure: Comparing conflict of interest policy among the United States, France, and Australia. Health Policy, 2018. 122(5): p. 509–518. doi: 10.1016/j.healthpol.2018.03.015 29605526

[6] Fabbri A., et al., Sunshine Policies and Murky Shadows in Europe: Disclosure of Pharmaceutical Industry Payments to Health Professionals in Nine European Countries. International Journal of Health Policy and Management, 2018. 7(6): p. 504–509. doi: 10.15171/ijhpm.2018.20 29935127 PMC6015505

[7] Ozaki A., et al., Overview and transparency of non-research payments to healthcare organizations and healthcare professionals from pharmaceutical companies in Japan: Analysis of payment data in 2016. Health Policy, 2020. 124(7): p. 727–735. doi: 10.1016/j.healthpol.2020.03.011 32439213

[8] Robbins N.M., Meyer M.J., and Bernat J.L., Scope and nature of financial conflicts of interest between neurologists and industry. 2013–2016, 2019. 93(10): p. 438–449. 10.1212/WNL.0000000000008067 31383793

[9] Ozaki A., et al., Pharmaceutical payments to certified oncology specialists in Japan in 2016: a retrospective observational cross-sectional analysis. BMJ open, 2019. 9(9): p. e028805–e028805. doi: 10.1136/bmjopen-2018-028805 31494604 PMC6731803

[10] Mulinari S. and Ozieranski P., Disclosure of payments by pharmaceutical companies to healthcare professionals in the UK: analysis of the Association of the British Pharmaceutical Industry’s Disclosure UK database, 2015 and 2016 cohorts. BMJ open, 2018. 8(10): p. e023094–e023094. doi: 10.1136/bmjopen-2018-023094 30344175 PMC6196800

[11] Mulinari S., et al., Pharmaceutical industry self-regulation and non-transparency: country and company level analysis of payments to healthcare professionals in seven European countries. Health Policy, 2021. doi: 10.1016/j.healthpol.2021.04.015 34006392

[12] Yeh J.S., et al., Association of Industry Payments to Physicians With the Prescribing of Brand-name Statins in Massachusetts. JAMA Internal Medicine, 2016. 176(6): p. 763–768. doi: 10.1001/jamainternmed.2016.1709 27159336

[13] Goupil B., et al., Association between gifts from pharmaceutical companies to French general practitioners and their drug prescribing patterns in 2016: retrospective study using the French Transparency in Healthcare and National Health Data System databases. BMJ, 2019. 367: p. l6015–l6015. doi: 10.1136/bmj.l6015 31690553 PMC6830500

[14] Mitchell A.P., et al., Are Financial Payments From the Pharmaceutical Industry Associated With Physician Prescribing? Annals of Internal Medicine, 2020. doi: 10.7326/M20-5665 33226858 PMC8315858

[15] Mejia J., Mejia A., and Pestilli F., Open data on industry payments to healthcare providers reveal potential hidden costs to the public. Nature Communications, 2019. 10(1): p. 4314. doi: 10.1038/s41467-019-12317-z 31541096 PMC6754508

[16] Davis C., Unhealthy Pharmaceutical Regulation: Innovation, Politics and Promissory Science. 1st ed. 2013. ed, ed. Abraham J.. 2013, London: London: Palgrave Macmillan UK: Imprint: Palgrave Macmillan.

[17] E.F.P.I.A., EFPIA Report on Ethics & Compliance Activities: June 2020. 2020, European Federation of Pharmaceutical Industries and Associations: Brussels. p. 33.

[18] Anderson T.S., Gellad W.F., and Good C.B., Characteristics Of Biomedical Industry Payments To Teaching Hospitals. Health Affairs, 2020. 39(9): p. 1583–1591. doi: 10.1377/hlthaff.2020.00385 32897778

[19] Ozieranski P., et al., Analysis of Pharmaceutical Industry Payments to UK Health Care Organizations in 2015. JAMA Network Open, 2019. 2(6): p. e196253–e196253. doi: 10.1001/jamanetworkopen.2019.6253 31225896 PMC6593961

[20] Moberly T., The pharma deals that CCGs fail to declare. BMJ, 2018. 360: p. j5915–j5915. doi: 10.1136/bmj.j5915 29298768

[21] NHS England, Clinical Commissioning Groups (CCGs). 2021; Available from: https://www.england.nhs.uk/commissioning/who-commissions-nhs-services/ccgs/-:~:text=CCGs%20are%20groups%20of%20general,for%20their%20patients%20and%20population.&text=CCGs%20are%20responsible%20for%20about,services%20(co%2Dcommissioning).

[22] Moberly T., NHS joint working with industry is out of public sight. BMJ (Clinical research ed.), 2019. 364: p. l1353–-l1353. doi: 10.1136/bmj.l1353 30917947

[23] Zosia K., Primary Care: A Century Of General Practice. BMJ: British Medical Journal, 2006. 332(7532): p. 39–40. doi: 10.1136/bmj.332.7532.39 16399738 PMC1325136

[24] Greer S.L., Devolution and health in the UK: policy and its lessons since 1998. British Medical Bulletin, 2016. 118(1): p. 16–24. doi: 10.1093/bmb/ldw013 27151953 PMC5127421

[25] NHS Digital, Patients Registered at a GP Practice July 2020. 2020, Available from: https://digital.nhs.uk/data-and-information/publications/statistical/patients-registered-at-a-gp-practice/july-2020.

[26] NHS England, Primary Care. 2020, Available from: https://www.england.nhs.uk/five-year-forward-view/next-steps-on-the-nhs-five-year-forward-view/primary-care/.

[27] Fleischman W., et al., Association between payments from manufacturers of pharmaceuticals to physicians and regional prescribing: cross sectional ecological study. BMJ, 2016. 354: p. i4189. doi: 10.1136/bmj.i4189 27540015 PMC4989280

[28] DeJong C., et al., Pharmaceutical Industry–Sponsored Meals and Physician Prescribing Patterns for Medicare Beneficiaries. JAMA Internal Medicine, 2016. 176(8): p. 1114–1122. doi: 10.1001/jamainternmed.2016.2765 27322350

[29] Duerden M., et al., The Quality of GP Prescribing. 2011, King’s Fund: London.

[30] Hajjar E.R., Cafiero A.C., and Hanlon J.T., Polypharmacy in elderly patients. The American Journal of Geriatric Pharmacotherapy, 2007. 5(4): p. 345–351. doi: 10.1016/j.amjopharm.2007.12.002 18179993

[31] Ozierański P., McKee M., and King L., Pharmaceutical lobbying under postcommunism: universal or country-specific methods of securing state drug reimbursement in Poland? Health Economics, Policy and Law, 2012. 7(2): p. 175–195. doi: 10.1017/S1744133111000168 21819633

[32] Frazer J.S. and Frazer G.R., GP prescribing in Northern Ireland by deprivation index: retrospective analysis. Family Medicine and Community Health, 2020. 8(3): p. e000376. doi: 10.1136/fmch-2020-000376 32565488 PMC7307529

[33] Covvey J.R., et al., An association between socioeconomic deprivation and primary care antibiotic prescribing in Scotland. Journal of Antimicrobial Chemotherapy, 2014. 69(3): p. 835–841. doi: 10.1093/jac/dkt439 24176983

[34] Angell M., The truth about the drug companies: how they deceive us and what to do about it. 2004, New York: New York: Random House.

[35] Sismondo S., Key Opinion Leaders and the Corruption of Medical Knowledge: What the Sunshine Act Will and Won’t Cast Light on. Journal of Law, Medicine & Ethics, 2013. 41(3): p. 635–643. doi: 10.1111/jlme.12073 24088154

[36] Ozieranski P. and King L.P., Governing drug reimbursement policy in Poland: The role of the state, civil society, and the private sector. Theory and Society, 2017. 46(6): p. 577–610.

[37] Ozierański P. and King L., The persistence of cliques in the post-communist state. The case of deniability in drug reimbursement policy in Poland: The persistence of cliques in the post-communist state. The British journal of sociology, 2016. 67(2): p. 216–241. doi: 10.1111/1468-4446.12193 27206533

[38] Hollands S., Receipt of Promotional Payments at the Individual and Physician Network Level Associated with Higher Branded Antipsychotic Prescribing Rates. Administration and policy in mental health, 2020. 47(1): p. 73–85. doi: 10.1007/s10488-019-00974-7 31515636 PMC7288218

[39] Zeltzer D. and Agha L., Drug Diffusion Through Peer Networks: The Influence of Industry Payments. 2019. 10.1257/pol.20200044 PMC9387671 35992019

[40] Sismondo S., Hegemony of Knowledge and Pharmaceutical Industry Strategy, in Philosophical Issues in Pharmaceutics: Development, Dispensing, and Use, Ho D, Editor. 2017, Springer Netherlands: Dordrecht. p. 47–63.

[41] Rahul A., et al., Leveraging CMS Open Payments Data to Identify Channel Preferences and Gather Competitive Intelligence, Thereby Improving HCP Targeting. 2021, Pharmaceutical Management Science Association.

[42] Oldani, M.J., Tales from the ’Script’: An Insider/Outsider View of Pharmaceutical Sales Practices. Kroeber Anthropological Society Papers, 2002: p. 147–176.

[43] Association of the British Pharmaceutical, I., Code of Practice for the Pharmaceutical Industry. 2019, Prescription Medicines Code of Practice Authority: London.

[44] NHS England, Friends and Family Test data–October 2016. 2016.

[45] Ministry of Housing, C.a.L.G. English Indices of Deprivation 2015. 2019 [cited 2019 20 November]; Available from: https://imd-by-postcode.opendatacommunities.org/imd/2015.

[46] Smith T., et al., The English Indices of Deprivation 2015: Technical Report. 2015, Department for Communities and Local Government: London.

[47] Team R.C., R: A language and environment for statistical computing, in R Foundation for Statistical Computing. 2018: Vienna.

[48] Borgatti S.P., Everett M.G., and Freeman L.C., UCINET. 2018, New York, NY: Springer New York: New York, NY. p. 3243–3249.

[49] Bastian, M., S. Heymann, and M. Jacomy, Gephi: An Open Source Software for Exploring and Manipulating Networks. Proceedings of the International AAAI Conference on Web and Social Media, 2009. 3(1).

[50] Borgatti S.P., Analyzing social networks. Second edition. ed, ed. Everett M.G. and Johnson J.C.. 2018, London: London: SAGE.

[51] Scott J., Social network analysis. 4th edition. ed. 2017, Los Angeles, Calif.: Los Angeles, Calif.: SAGE.

[52] Hanneman, R.A. and M. Riddle. Introduction to social network methods. 2005, Available from: http://faculty.ucr.edu/~hanneman/.

[53] Lo B., et al., Conflict of interest in medical research, education, and practice. 2009, Washington, DC: Washington, DC: National Academies Press. 20662118

[54] Hadland S.E., et al., Association of Pharmaceutical Industry Marketing of Opioid Products With Mortality From Opioid-Related Overdoses. JAMA Network Open, 2019. 2(1): p. e186007–e186007. doi: 10.1001/jamanetworkopen.2018.6007 30657529 PMC6484875

[55] Annapureddy A.R., et al., Association Between Industry Payments to Physicians and Device Selection in ICD Implantation. Jama, 2020. 324(17): p. 1755–1764. doi: 10.1001/jama.2020.17436 33141208 PMC7610190

[56] P.M.C.P.A., Code of Practice for the Pharmaceutical Industry 2015. 2015, Association of the British Pharmaceutical Industry: London. p. 69.

[57] Rickard E., Ozieranski P., and Mulinari S., Evaluating the transparency of pharmaceutical company disclosure of payments to patient organisations in the UK. Health Policy, 2019. 123(12): p. 1244–1250. doi: 10.1016/j.healthpol.2019.08.007 31455562

[58] Mintzes B., Should patient groups accept money from drug companies? No. BMJ, 2007. 334(7600): p. 935–935. doi: 10.1136/bmj.39185.394005.AD 17478846 PMC1865416

[59] Katz, D., J.F. Caplan Al Fau—Merz, and J.F. Merz, All gifts large and small: toward an understanding of the ethics of pharmaceutical industry gift-giving. (1536–0075 (Electronic)). 10.1162/15265160360706552 14594489

[60] Krimsky, S., Small gifts, conflicts of interest, and the zero-tolerance threshold in medicine. (1536–0075 (Electronic)). 10.1162/15265160360706589 14594492

[61] Dana J. and Loewenstein G., A Social Science Perspective on Gifts to Physicians From Industry. JAMA, 2003. 290(2): p. 252–255. doi: 10.1001/jama.290.2.252 12851281

[62] Marks J.H., The Perils of Partnership: Industry Influence, Institutional Integrity, and Public Health. 2019, New York: Oxford University Press. 250.

[63] Zetterqvist A.V. and Mulinari S., Misleading Advertising for Antidepressants in Sweden: A Failure of Pharmaceutical Industry Self-Regulation. PLOS ONE, 2013. 8(5): p. e62609. doi: 10.1371/journal.pone.0062609 23650519 PMC3641086

[64] Zetterqvist A.V., Merlo J. and Mulinari S., Complaints, Complainants, and Rulings Regarding Drug Promotion in the United Kingdom and Sweden 2004–2012: A Quantitative and Qualitative Study of Pharmaceutical Industry Self-Regulation. PLoS Med, 2015. 12(2): p.e1001785. doi: 10.1371/journal.pmed.1001785 25689460 PMC4331559

[65] P.M.C.P.A., Code of Practice for the Pharmaceutical Industry 2019. 2019, Association of the British Pharmaceutical Industry: London. p. 69.

[66] P.M.C.P.A. Completed Cases. 2021 [cited 2021 04/03/2021]; Available from: https://www.pmcpa.org.uk/cases/completed-cases/.

[67] Ho K.H., van Hove M., and Leng G., Trends in anticoagulant prescribing: a review of local policies in English primary care. BMC health services research, 2020. 20(1): p. 279–279. doi: 10.1186/s12913-020-5058-1 32245380 PMC7126454

[68] Wilcock M., Pharmaceutical marketing—greater than the sum of its parts? Drug and therapeutics bulletin, 2020. 58(10): p. 147–149. doi: 10.1136/dtb.2020.000004 32826309

[69] England N., Managing Conflicts of Interest in the NHS: Guidance for Staff and Organisations. 2017, NHS England: London. p. 33.

[70] Parker L., et al., ‘Lines in the sand’: an Australian qualitative study of patient group practices to promote independence from pharmaceutical industry funders. BMJ Open, 2021. 11(2): p. e045140. doi: 10.1136/bmjopen-2020-045140 33563626 PMC7875302

[71] Greenway T. and Ross J.S., US drug marketing: how does promotion correspond with health value? BMJ, 2017. 357: p. j1855. doi: 10.1136/bmj.j1855 28465309

[72] Rimmer A., Briefing: Why do we need a mandatory register of doctors’ interests? BMJ, 2021. 373: p. n1280. doi: 10.1136/bmj.n1280 34016582

[73] Lexchin J. and Fugh-Berman A., A Ray of Sunshine: Transparency in Physician-Industry Relationships Is Not Enough. Journal of General Internal Medicine, 2021. 36(10): p. 3194–3198. doi: 10.1007/s11606-021-06657-0 33694070 PMC8481515

[74] Kanter G.P., et al., Effect of the public disclosure of industry payments information on patients: results from a population-based natural experiment. BMJ Open, 2019. 9(2): p. e024020. doi: 10.1136/bmjopen-2018-024020 30826793 PMC6398799

