# Supplementary material for: Drug company payments to General Practices in England: Cross-sectional and social network analysis
Source: PLoS One. 2021 Dec 7;16(12):e0261077. doi: 10.1371/journal.pone.0261077 (PMC8651134; doi:10.1371/journal.pone.0261077)
Supplement: S1 Appendix — (DOCX) [file pone.0261077.s001.docx]

S1 Appendix – Coding of general practices

Healthcare organsiations coded as "Primary care provieder organisations" in Ozieranski P, Csanadi M, Rickard E, Tchilingirian J, Mulinari S. Analysis of Pharmaceutical Industry Payments to UK Health Care Organizations in 2015. JAMA Netw Open. 2019;2(6):e196253. doi:10.1001/jamanetworkopen.2019.6253

1. general practice, surgery, medical practice or family practice
2. health centre, medical centre or primary care centre
3. out-of-hours service (OOH) - primary care
4. healthcare or medical group
5. group of surgeries or medical practices
6. federation, alliance, association, federation or consortium of general practices
7. collaboratives of primary care organisations

Healthcare organsiations coded as general practices

1. general practice, surgery, medical practice or family practice
2. health centre, medical centre or primary care centre
