# Supplementary material for: Drug company payments to General Practices in England: Cross-sectional and social network analysis
Source: PLoS One. 2021 Dec 7;16(12):e0261077. doi: 10.1371/journal.pone.0261077 (PMC8651134; doi:10.1371/journal.pone.0261077)
Supplement: S2 Appendix — (DOCX) [file pone.0261077.s002.docx]

S2 Appendix – Data cleaning protocol

**Data cleaning and merging process**

1. **manual assignment of practice codes to to practices in main dataset (Disclosure UK, 2015)**
   - practice codes taken from NHS’s Friends and Family Test Data collected in 2015
   - <https://www.england.nhs.uk/statistics/statistical-work-areas/friends-and-family-test/friends-and-family-test-data/>
   - this dataset has information on practice codes, post codes, GP practice name and GP practice address, which was compared with the Disclosure UK dataset (hence the need for manual matching)
   - some postcode changes were done on the Disclosure UK dataset after additional internet search
     - line 739: B44 0NN changed to RM3 7JJ
     - line 2425: BD15 7NJ changed to BD15 7WA
       - <https://www.cqc.org.uk/location/1-540428462/contact>
     - line 2426: BN21 1RT changed to BN21 1RR
       - <https://www.nhs.uk/Services/GP/Overview/DefaultView.aspx?id=36869>
     - line 2947: BS13 8QB changed to BS13 8QA
       - <https://www.nhs.uk/services/clinics/mapsanddirections/defaultview.aspx?id=115015>
     - lines 1232, 1233, 1234: practice code and postcode doesn’t match but the practice is the same, it just has several places
       - <https://www.nhs.uk/Services/GP/Overview/DefaultView.aspx?id=43748>
     - line 2191: practice code and postcode doesn’t match but the practice is the same
   - number of GP practices in Disclosure UK dataset without identifiable practice codes: 149
     - these had to be excluded from the dataset
     - random distribution of the GP practices across England is demonstrated on a map (see map in appendix)
   - sample size at this stage: 2797
2. **merging on Disclosure UK dataset (now with new practice_code variable) to the Patients Registered at a GP Practice NHS dataset from 2015**
   - <https://digital.nhs.uk/data-and-information/publications/statistical/patients-registered-at-a-gp-practice>
   - many to one merge in Stata
3. **merging the now merged new dataset with another dataset showing multiple deprivation indices for the postcodes in new dataset**
   - data taken from the website of the Ministry of Housing, Communities and Local Government
   - <http://imd-by-postcode.opendatacommunities.org/imd/2019>
4. **summing up the all the transfer of values (TOVs) per GP practices**
   - reducing data to a dataset with one observation per ID containing the total TOV
   - code used in Stata: collapse (sum) TOVs, by(Practice_code)
   - new dataset with only two variables: Practice_code and TOVs
5. **merging total TOVs 2-variable dataset with original merged dataset creating the final dataset with all variables included and reduced to one row per each GP practice with sum of TOV**
   - one-to-many merging
   - final sample size: 1643
