# Supplementary material for: Drug company payments to General Practices in England: Cross-sectional and social network analysis
Source: PLoS One. 2021 Dec 7;16(12):e0261077. doi: 10.1371/journal.pone.0261077 (PMC8651134; doi:10.1371/journal.pone.0261077)
Supplement: S3 Appendix — (DOCX) [file pone.0261077.s003.docx]

## S3 Appendix. Total value and number of payments to the top 10 healthcare organisations in England in 2015

| HCO categories | Value of payments (£) | Number of payments |
| --- | --- | --- |
| University | 9,040,463.73 | 869 |
| NHS Foundation Trust | 8,813,525.32 | 4113 |
| NHS Trust | 4,026,402.19 | 2198 |
| Multi-professional organisation) | 3,484,753.15 | 541 |
| General practices | 2,726,017.77 | 2945 |
| Medical training or education services | 1,641,017.95 | 828 |
| Multi-professional organisation - different healthcare professionals | 1,628,235.57 | 326 |
| Multipurpose charity | 1,090,917.52 | 231 |
| Private company providing other health services | 873,565.81 | 22 |
| Clinical Commissioning Group (CCG) - England | 830,988.64 | 1842 |

Notes: The categorisation of healthcare organisations is based on unpublished data (Anonymous)
