# Supplementary material for: Drug company payments to General Practices in England: Cross-sectional and social network analysis
Source: PLoS One. 2021 Dec 7;16(12):e0261077. doi: 10.1371/journal.pone.0261077 (PMC8651134; doi:10.1371/journal.pone.0261077)
Supplement: S4 Appendix — (DOCX) [file pone.0261077.s004.docx]

S4 Appendix – Distribution of practices across different regions England

| Regions | Practices receiving payments used in the analysis (% of total receiving payments) | Total practices | Percentage of practices receiving payments (out of total practices) | Number of excluded practices from analysis | Percentage of excluded practices (out of practices used in the analysis) | Percentage of excluded practices from analysis (out of total number of practices) |
| --- | --- | --- | --- | --- | --- | --- |
| East Midlands | 147 (8.95%) | 751 | 19.57 % | 15 | 10.20 % | 2.00 % |
| East of England | 136 (8.28%) | 706 | 19.26 % | 17 | 12.50 % | 2.41 % |
| London | 140 (8.62%) | 1576 | 8.88 % | 15 | 10.71 % | 0.95 % |
| North East England | 107 (6.51%) | 730 | 14.66 % | 23 | 21.50 % | 3.15 % |
| North West England | 261 (15.89%) | 1352 | 19.30 % | 19 | 7.28 % | 1.41 % |
| South East England | 249 (15.16%) | 937 | 26.57 % | 16 | 6.43 % | 1.71 % |
| South West England | 168 (10.23%) | 703 | 23.90 % | 22 | 13.10 % | 3.13 % |
| West Midlands | 220 (13.39%) | 931 | 23.63 % | 20 | 9.09 % | 2.15 % |
| Yorkshire and the Humber | 215 (13.09%) | no data | no data | no data | no data | no data |
| Total | 1643 | 7686 | 21.38 % | 147 | 8.95 % | 1.91 % |

Notes: We did not find data on the number of practices in Yorkshire and the Humber. These practices are possibly counted together with practices in North East England. This table is based on Disclosure UK (2015, version 20160630).
