# Supplementary material for: Drug company payments to General Practices in England: Cross-sectional and social network analysis
Source: PLoS One. 2021 Dec 7;16(12):e0261077. doi: 10.1371/journal.pone.0261077 (PMC8651134; doi:10.1371/journal.pone.0261077)
Supplement: S5 Appendix — (DOCX) [file pone.0261077.s005.docx]

## S5 Appendix. Breakdown of payments types received by general practices

| Payment types | Total number of payments (% of total) | Sum value of payments (% of total) | Number of practices |
| --- | --- | --- | --- |
| Contribution to costs of events | 1,006 (34.16) | £613,505.98 (22.51) | 417 |
| Donations and grants to HCOs | 1,890 (64.18) | £2,081,553.19 (76.36) | 1,349 |
| Fee for service and consultancy | 49 (1.66) | £30,958.61 (1.14) | 27 |
| Total | 2,945 | £2,726,017.78 | 1,643 |

Notes: Data refers to the original Disclosure UK dataset before exclusions. This table is based on Disclosure UK (2015, version 20160630).
