# Supplementary material for: Drug company payments to General Practices in England: Cross-sectional and social network analysis
Source: PLoS One. 2021 Dec 7;16(12):e0261077. doi: 10.1371/journal.pone.0261077 (PMC8651134; doi:10.1371/journal.pone.0261077)
Supplement: S6 Appendix — (DOCX) [file pone.0261077.s006.docx]

## S6 Appendix - Payments to the top ten general practices

| Top practices | Region | Total value of payments (£) | Number of payments | Number of donors | Minimum value | Median value [IQR] | Maximum value |
| --- | --- | --- | --- | --- | --- | --- | --- |
| 1 | North West | 148,395.20 | 22 | 5 | 160.00 | 8,123.00 [6,194.00] | 11,675.00 |
| 2 | London | 52,773.80 | 2 | 2 | 3,353.00 | 26,387.00 [23,034.00] | 49,421.00 |
| 3 | South West | 32,827.16 | 132 | 18 | 35.12 | 200.00 [160.00] | 900.00 |
| 4 | East of England | 28,420.99 | 41 | 16 | 33.00 | 167.00 [120.00] | 18,600.00 |
| 5 | North East | 24,000.00 | 1 | 1 | 24,000.00 | 24,000.00 [0.00] | 24,000.00 |
| 6 | South East | 20,831.00 | 1 | 1 | 20,831.00 | 20,831.00 [0.00] | 20,831.00 |
| 7 | Yorkshire and the Humber | 18,976.80 | 1 | 1 | 18,977.00 | 18,977.00 [0.00] | 18,977.00 |
| 8 | North West | 15,200.00 | 1 | 1 | 15,200.00 | 15,200.00 [0.00] | 15,200.00 |
| 9 | North West | 15,131.36 | 2 | 1 | 203.40 | 7,565.70 [7,362.30] | 14,928.00 |
| 10 | North West | 14,705.60 | 2 | 1 | 1,914.00 | 7,353.00 [5,439.00] | 12,792.00 |

Notes: This table is based on Disclosure UK (2015, version 20160630), the GP Friends and Family Test (FFT) dataset, and the Patients Registered at a GP Practice 2015 NHS dataset. The Practice Codes refer to individual practices.
