# Supplementary material for: Drug company payments to General Practices in England: Cross-sectional and social network analysis
Source: PLoS One. 2021 Dec 7;16(12):e0261077. doi: 10.1371/journal.pone.0261077 (PMC8651134; doi:10.1371/journal.pone.0261077)
Supplement: S7 Appendix — (DOCX) [file pone.0261077.s007.docx]

## S7 Appendix - Summary of network statistics calculated for valued networks of drug companies

| Network measures | All payments | Payments per general practice worth at least £100 | Payments per general practice worth at least £1,000 | Payments per general practice worth at least £2,500 |
| --- | --- | --- | --- | --- |
| Centralisation | 0.2112 | 0.1981 | 0.0697 | 0.0403 |
| Density | 1.627 | 1.453 | 0.146 | 0.036 |
| Companies with the highest centrality score | Bayer (0.242) | Bayer  (0.229) | Bayer  (0.076) | Eli Lilly  (0.047) |
|  |  |  |  |  |
|  | **All payments** | **Over 1 payment to a single** practice | **Over 5 payments to a single** practice | **Over 10 payments to a single** practice |
| Centralisation | 0.2112 | 0.1752 | 0.1069 | 0.1361 |
| Density | 1.627 | 0.396 | 0.049 | 0.028 |
| Companies with the highest centrality score | Bayer (0.242) | Takeda  (0.244) | Chiesi  (0.125) | Takeda  (0.156) |

Notes: This table shows statistics calculated for networks of companies established by making payemnts to the same practices. The networks are “valued”, which means that the making payemnts to the same practices. The networks are “valued”, which means that they consdider the number of “shared” practices not only the fact of sharing practices. The results of analysis based on the value of payments are presented in the upper part of the table, while those based on the number of payments – in the lower part of the table. This table is based on Disclosure UK (2015, version 20160630).
