# Supplementary material for: Drug company payments to General Practices in England: Cross-sectional and social network analysis
Source: PLoS One. 2021 Dec 7;16(12):e0261077. doi: 10.1371/journal.pone.0261077 (PMC8651134; doi:10.1371/journal.pone.0261077)
Supplement: S8 Appendix — (DOCX) [file pone.0261077.s008.docx]

## S8 Appendix - Network visualizations for networks based on the number of payments

Notes: a) network of all payments; b) network of over 1 payment per surgery; c) network of over 5 payments per surgery; d) network of over 10 payments per surgery. Figures a-d shows the visualisation of networks based on the number of payments, created in Gephi. Node label size and darkness corresponds to the centrality of a company, the strength and darkness of the lines corresponds to the number of shared practices between companies. The networks visibly change as the payment number to a single surgery increases.
