# Supplementary material for: Drug company payments to General Practices in England: Cross-sectional and social network analysis
Source: PLoS One. 2021 Dec 7;16(12):e0261077. doi: 10.1371/journal.pone.0261077 (PMC8651134; doi:10.1371/journal.pone.0261077)
Supplement: S9 Appendix — (DOCX) [file pone.0261077.s009.docx]

## S9 Appendix. Breakdown of network statistics according to general practice characteristics

### Results for valued networks by number of registered patients, proportion of elderly patients and MDI

| Network measures | Payments to practices by the number of registered patients (1st Quartile) | Payments to practices by the number of registered patients (2nd Quartile) | Payments to practices by the number of registered patients (3rd Quartile) | Payments to practices by the number of registered patients (4th Quartile) |
| --- | --- | --- | --- | --- |
| Centralisation | 0.119 | 0.124 | 0.233 | 0.198 |
| Density | 0.053 | 0.125 | 0.316 | 1.133 |
| Standard deviation | 0.365 | 0.550 | 1.130 | 2.190 |
| Companies with the highest centrality score | Bayer (16/0.125) | Bayer (30/0.134) | Bayer (80/0.250) | Bayer (161/0.240) |
|  | **Payments to practices by the proportion of elderly patients (1^st^ Quartile)** | **Payments to practices by the proportion of elderly patients (2^nd^ Quartile)** | **Payments to practices by the proportion of elderly patients (3^rd^ Quartile)** | **Payments to practices by the proportion of elderly patients (4^th^ Quartile)** |
| Centralisation | 0.193 | 0.106 | 0.235 | 0.175 |
| Density | 0.722 | 0.186 | 0.386 | 0.333 |
| Standard deviation | 1.193 | 0.781 | 1.099 | 1.194 |
| Companies with the highest centrality score | Bayer (81/0.253) | Bayer (44/0.115) | Bayer (83/0.259) | Bayer (79/0.190) |
|  | **Payments to practices by MDI (1^st^ Quartile)** | **Payments to practices by MDI (2^nd^ Quartile)** | **Payments to practices by MDI (3^rd^ Quartile)** | **Payments to practices by MDI (4^th^ Quartile)** |
| Centralisation | 0.188 | 0.140 | 0.142 | 0.184 |
| Density | 0.777 | 0.176 | 0.284 | 0.390 |
| Standard deviation | 1.254 | 0.833 | 1.126 | 1.179 |
| Companies with the highest centrality score | Bayer (87/0.247) | Bayer (52/0.148) | Bayer (69/0.154) | Bayer (79/0.206) |

Notes: Network statistics were calculated in UCInet.

### Results for valued networks by regions

| Network measures | East Midlands | East of England | London | North East England | North West England | South East England | South West England | West Midlands | | Yorkshire and the Humber |
| --- | --- | --- | --- | --- | --- | --- | --- | --- | --- | --- |
| Centralisation | 0.136 | 0.152 | 0.103 | 0.141 | 0.172 | 0.090 | 0.181 | 0.103 | 0.114 | |
| Density | 0.055 | 0.275 | 0.136 | 0.117 | 0.390 | 0.076 | 0.434 | 0.104 | 0.040 | |
| Standard deviation | 0.287 | 0.546 | 0.508 | 0.490 | 1.343 | 0.372 | 0.776 | 0.563 | 0.276 | |
| Companies with the highest centrality score | Bayer (14/0.146) | Bayer (27/0.211) | Bayer (23/0.120), Eli Lilly (23/0.120) | Bayer (25/0.156) | Bayer (90/0.188) | Bayer (16/0.100), Eli Lilly (16/0.100) | Bayer (52/0.232) | Bayer (25/0.112) | Bayer (15/0.117) | |

Notes: Network statistics were calculated in UCInet.

### c) Results for binary networks by number of registered patients, proportion of elderly patients and MDI

| Network measures | Payments to practices by the number of registered patients  (1st Quartile) | Payments to practices by the number of registered patients (2nd Quartile) | Payments to practices by the number of registered patients (3rd Quartile) | Payments to practices by the number of registered patients (4th Quartile) |
| --- | --- | --- | --- | --- |
| Centralisation | 0.1648 | 0.3087 | 0.3958 | 0.3826 |
| Density | 0.027 | 0.073 | 0.143 | 0.428 |
| Standard deviation | 0.161 | 0.260 | 0.350 | 0.495 |
| Companies with the highest centrality score | Bayer (6/0.182) | Eli Lilly (12/0.364) | Bayer (17/0.515) | Bayer (26/0.788) |
|  | **Payments to practices by the proportion of elderly patients (1st Quartile)** | **Payments to practices by the proportion of elderly patients (2nd Quartile)** | **Payments to practices by the proportion of elderly patients (3rd Quartile)** | **Payments to practices by the proportion of elderly patients (4th Quartile)** |
| Centralisation | 0.3314 | 0.3731 | 0.4621 | 0.3314 |
| Density | 0.385 | 0.103 | 0.201 | 0.143 |
| Standard deviation | 0.487 | 0.304 | 0.401 | 0.350 |
| Companies with the highest centrality score | Bayer (23/0.697), Eli Lilly (23/0.697), Sanofi Aventis (23/0.697) | Bayer (15/0.455) | Bayer (21/0.636) | Boehringer Ingelheim (15/0.455) |
|  | **Payments to practices by MDI (1st Quartile)** | **Payments to practices by MDI (2nd Quartile)** | **Payments to practices by MDI (3rd Quartile)** | **Payments to practices by MDI (4th Quartile)** |
| Centralisation | 0.3485 | 0.2955 | 0.3750 | 0.3826 |
| Density | 0.399 | 0.086 | 0.132 | 0.185 |
| Standard deviation | 0.490 | 0.280 | 0.338 | 0.389 |
| Companies with the highest centrality score | Bayer (24/0.727) | Bayer (12/0.364) | Bayer (16/0.485) | Bayer (18/0.545) |

Notes: Network statistics were calculated in UCInet.

### Results for binary networks by region

| Network measures | East Midlands | East of England | London | North East England | | North West England | | South East England | South West England | West Midlands | Yorkshire and the Humber |
| --- | --- | --- | --- | --- | --- | --- | --- | --- | --- | --- | --- |
| Centralisation | 0.2140 | 0.2765 | 0.2879 | 0.3409 | 0.4659 | | 0.2367 | | 0.3693 | 0.3277 | 0.2614 |
| Density | 0.041 | 0.225 | 0.093 | 0.073 | 0.168 | | 0.050 | | 0.319 | 0.055 | 0.027 |
| Standard deviation | 0.198 | 0.417 | 0.290 | 0.260 | 0.373 | | 0.218 | | 0.466 | 0.228 | 0.161 |
| Companies with the highest centrality score | Bayer (8/0.242) | Bayer (16/0.485), Boehringer Ingelheim (16/0.485), Novo Nordisk (16/0.485), Sanofi Aventis (16/0.485) | Eli Lilly (12/0.364) | Bayer (13/0.394) | Bayer (20/0.606) | | Eli Lilly (9/0.273) | | Takeda (22/0.667) | Bayer (12/0.364) | Bayer (9/0.273) |

Notes: Network statistics were calculated in UCInet.
