# Supplementary material for: Drug company payments to General Practices in England: Cross-sectional and social network analysis
Source: PLoS One. 2021 Dec 7;16(12):e0261077. doi: 10.1371/journal.pone.0261077 (PMC8651134; doi:10.1371/journal.pone.0261077)
Supplement: Attachment — Submitted filename: Addressing comments_ES_30.09_final.docx [file pone.0261077.s010.docx]

| **Editor’s comments** | **Authors’ response** |
| --- | --- |
| **File naming**: Please ensure that your manuscript meets PLOS ONE's  style requirements, including those for file naming. (link in the email) | We have followed the PLOS One’s style requrements. |
| **Competing interests section**: Please confirm that this does not alter your adherence to all PLOS ONE policies on sharing data and materials, by including the following statement: "This does not alter our adherence to PLOS ONE policies on sharing data and materials.”  Please include your updated Competing Interests statement in your cover letter; we will change the online submission form on your behalf. | We can confirm we have not altered our adherence to all PLOS ONE’s policies and have included the requested sentence. |

|  | **Reviewer 1’s comments** | **Authors’ response** |
| --- | --- | --- |
| 1 | In terms of the overall framing and interpretation of study results, it would be good to know how this relates to funding of individual GPs who are working within these surgeries. Is a link possible? If not, it would be good to relate this funding to total GP funding for 2015 (if data are available) and to consider this in the overall framing / interpretation of the study results. | Thank you for this important question. We know, primarily from the US research informed by the Open Payments Database, that drug company payments to physicians cause concerns regarding conflicts of interest and can lead to the prescription of higher cost drugs. However, unlike in the US, physicians in most European countries following the self-regulatory approach to disclosure are allowed to refuse the disclosure of the payments they received, in line with the drug company interpretation of European privacy laws by drug company trade groups.  In response to your comments, in the introduction we now stress the non-disclosure issue in Europe (p. 3, para 3), including specifically in reation to the UK. We also mention the lack of individual-level analysis as a limitation of our research in the Discussion (p. 22, para 3). |
| 2 | I am also unsure what the network analysis adds to understanding of either companies’ payment patterns or surgeries receptivity to payments. Its value to answer clear research questions on funding patterns and purpose wasn't clear. The assumption of competing companies (if they are funding the same surgery) may be incorrect, as without information about which products each company is promoting, they might or might not be competing (e.g. may be promoting in a different treatment area). | Thank you for helping us clarify our argument. We have justified the importance of SNA in the Introduction (p. 5, para 2).  In responding to your concerns re the use of SNA, we would like to note separately that social network analysis considers the structure of connections between companies and practices, rather than the distribution of payments like standard descriptive analyses. By connecting companies paying the same practices, we can map out the structure of payment network and see:  a) which companies are interested in the same practices,  b) if some companies are dominating the payment networks,  c) the differential structures of networks for different values of payments.  This is a novel way to look at industry payments in the healthcare sector and, although we can’t come to definite conclusions based on this analysis, partly due to the lack of individual-level data available, we can prompt new research questions and new ways to analyse disclosure data. |
| 3 | If you wanted to provide some information to readers about the pattern of company payments based on the network analysis, I would suggest just including S6(a), the figure with the network of all payments as a figure in your main report, and to explain what it shows about links between companies (ie darker lines and thicker lines mean more shared funding of the same GP surgery). Ideally you would use the concepts of 'centralisation' and 'density' as a guide for yourself for the extent of joint funding and of concentration of funding, but you would leave these jargon terms completely out of the analysis. I would leave out all of the tables and text related to this as well and just keep a very brief section on the results of the network analysis, presented in a way a general medical reader can understand. If you wanted to support this with the details of the network analysis results, this could be included in the supplement. | Thank you for this helpful suggestion. The original Appendix S6(b), showing network graphs based on the value of payments, has been added to the text and the network analysis results section has been rewritten, explaining what we can see from the graph. Alongside this, Table 4 with the centrality measured has been moved to the Appendices.  We kept the concepts of centrality, density and centralisation since these are essential for network analysis but they have been explained in a much simpler way with the help of the graphs and specifically in relation to the contex of our data. |
| 4 | Page 4: please add descriptive info on surgeries - # doctors on average per surgery and extent of variation, # of GP surgeries in total in the UK, if info is available. Also the first time ‘GP surgery’ is used it should likely be translated for North American audiences, where it’s called a GP practice. | We agree that it would be importat to include this data but, unfortunately, this is not possible.  There is NHS data on the number of GPs per practice but it is not complete for 2015, only data from some regions can be downloaded. Website: <https://www.nhsbsa.nhs.uk/prescription-data/catalyst-public-insight-portal>  However, we had to consider the GP practice data for 2015 because this is the only year for which we have the drug company payment data coded in a way that allow for isolating payments made to GP surgeries. Such coding is a very difficult and lengthy process.  Instead of data on doctors, we have indicators, such as the number of patients registered per practice, which arguably is a better indicator, since it is not as flux as the number of staff.  We have also renamed “surgeries” to “practices” consistently throughout the whole text, including tables and appendices.  Finally on this point, we would just like to point out that the focus of the study is England and not the UK. |
| 5 | Page 5: note about the strategic importance of surgeries needs some qualification. One complication in terms of surgeries being a key target of company payments is that individual GPs working within these surgeries would be a target for payments as well. From previous research on the UK transparency data, can you say anything about the proportion of payments in total going to GPs versus to hospital-based consultants? | Thank you for this important comment. We entirely agree that individual GPs are indeed probably a key target of funding. However, it is impossible to say if individuals or organisations are more important given the nature of the disclosed payment data that has been made available, especially the incompleteness of individual-level data given the issue of recipient non-disclosure, which was described in reference 10 (Mulinari and Ozieranski, 2019). This is also a wider problem of the approach to disclosure prevalent in European countries relying on industry self-regulation (need to cite Shai’s latest article here)  Therefore, in the introduction (p.3 para 3) we explain why individual-level research is impossible and why our organisational-level research gives the best and only possible insight into drgu company payment patterns. |
| 6 | Page 6, methods: It’s unclear whether the focus on products that are marketed unethically will provide the information needed, as these are not necessarily the most frequently marketed products; they could be a small subset of marketed products to GPs by each company. If data are available from IQVIA or other market research companies on product-specific advertising spending in the UK over the study time period, this would be much more relevant. IQVIA has provided this type of information to academic researchers free of charge in the past (in other countries). If this information was available then a second step would be to develop a list of the subset of drugs that would be used in primary care, based on a review by a clinician or frequency of use data if available. | We thank you for this comment. Please note that our analysis of PMCPA cases was an attempt to provide insight into the striking dominance of one company, Bayer More specifically, we wanted to get a clue into what product(s) Bayer’s payments could be associated with. Unfortunately, this is not reported in Disclosure UK and we have not been able to find product-specific marketing information for England or the UK from IQVIA or any other drug industry data analysis firm. Neither are we aware of any analysis based on such data to have ever been published.  However, we understand that our analysis is too “indirect” and agree that is far from perfect. We have therefore deleted this analysis from the paper. However, consistent with the suggestion below for the reviewer, we have kept some of the speculations about Bayer’s payments being related to rivaroxaban in the Discussion and also used it to highlight how marketing to GPs and GP practices may be problematic. |
| 7 | Page 7, methods: The decision to focus on 2015 is based on your team’s previous analysis [ref 17]. You had identified five payment categories to healthcare organisations in that study [listed in the Supplement]. Are these 5 types of payments included in the current study or a subset? Additionally eTable1 lists how the various type of health care organisations are classified in that previous article. It would be good to add a reference to how the GP surgeries were classified (for example if combined with other public sector health care providers, state this). | Thank you for your kind suggestion regarding the classification of GP surgeries. In the new Appendix S1 (to which we now refer in the Methods) we explain which categories of healthcare organisations exactly were coded as GP surgeries. The appendix also shows how this recipient category fits within the broader category of “Primary care provider organisations” reported in our previous paper (Ozieranski et al., 2019 [ref. no. 17 in article]). To answer your question specifically, therefore, the GP practices that we refer to in this paper is a nnarrower category than the one reported in the previous paper because we only wanted to focus on GP surgeries and not their federations or similar.  Payment categories and the share of payments and practices across these categories are stated in S4 Appendix. |
| 8 | Page 8: You note that specific surgeries were excluded from the analysis because of an inability to match the practice code with the payment data (6.7% of total payments). I’m wondering whether all parts of your analysis required this extra information, or only specific subsets in which you were looking at the characteristics of the GP surgeries. For example, when looking at the amount each company provided to GP surgeries in total, wouldn’t it be more accurate to include the entire set? | Matching payments with practice codes by addresses and surgery names was essential to confirm that a payment was in fact made to a practice (in other words, we used the address information to corroborate that the recipient was indeed a surgery). We cannot confirm that those 6.7% of payments without practice codes were made to surgeries if we include them in the analysis without finding them in the registry of GP surgeries. We also cannot confirm that some payments are made to different surgeries or the same surgery just written differently. For this reason, we completely excluded those payments from the analysis. Our preference was not to overestimate the number of GP surgieres. Also, we wanted to ensure that the numbers of GP surgeries and payments were the same in different tables. |
| 9 | Page 8-9: as noted above, I have some concerns about the selection of PMCPA investigations as an information source for which drugs were associated with payments to GP surgeries. You mention that this is likely the tip of the iceberg of payments. I would agree, but an additional concern is that it may not even necessarily represent the specific payment subset that focused on GP surgeries. In my opinion, this is too much of a stretch in terms of a link between this subset of payments and this subset of Disclosure UK payments. If you wanted to obtain a more accurate idea of which drugs were being most heavily promoted in 2015, my recommendation would be to try to obtain information on top drugs by company for advertising spending instead, and assess which are drugs used in primary care, as a more reliable information source on the focus of this funding of GP surgeries. | Thank you for this comment. Please see our response to point 6 which also addresses this comment. |
| 10 | Page 9: analyses: it is interesting to examine the number of companies providing payments to each surgery and the extent to which specific companies tend to co-fund specific surgeries, which I assume is the main focus of the network analysis. I think this provides an additional very interesting focus on patterns of payments.  If I understand centralisation it measures the extent to which a set of GP surgeries with similar characteristics (regionally or by patient population?) might obtain funding from a single company. I don’t really understand this statement in terms of what it measures of importance concerning industry funding of surgeries: “Centralisation score is measured as the ratio of the actual sum of centrality score differences and all possible sum of centrality score differences [53].”  Similarly, the measure of density as a proportion of existing ties between actors “as a share of all possible ties” needs to be explained in terms of the meaning to this specific study. It is not clear to me how this measures the “average strength of ties between companies”. It may be a situation in which companies use similar strategies to target specific GP practices, for example selecting those that have high prescribing rates and/or a patient population that is likely to use the promoted drug (e.g. if a drug for older people, a high enough proportion of older patients). Some of these marketing decisions might be directly competitive; others might simply be targeting the same surgeries for shared reasons but in a different treatment area. | The network analysis section has been presented in a simpler way, focusing on the value of payments only. Centrality, density and centralisation has been explained better with the help of the network graphs, which have been added to the text. However, in *Results: Decriptive analysis of the distribution of payments*, we have kept the description including the number of payments because these results are not difficult for the reader to interpret and they are essential in supporting our point on differential payment strategies (p.21, para 2). |
| 11 | P11: top of page – in describing the payments to surgeries and a proportion of total payments to healthcare organisations, it would be helpful to briefly state what types of other healthcare organisations received most of the funds. See below - the fact that they are 5th in payment level among healthcare organisations should be mentioned in results, not discussion.  Without this extra information, the latter seems inaccurate: “Consistent with our expectations, this finding suggests that surgeries were an important target of drug company payments within England’s healthcare system but their significance was reflected more by the monetary value of payments intensity of interactions, indicated by the number of payments.” I would suggest qualifying as the number of interactions is small (0.11% of total) and monetary value needs to be seen in context, eg. likely much less than payments to individuals. | We stated the HCOs that received the most payments and mentioned that practices were the 5^th^ on the list. We kept the proportion of the value of payments made to surgeries and deleted the proportion of the number of payments, since we shifted our focus to the value of payments and now the sentence is clearer (p. 10, para 2). |
| 12 | P11: fees for consultancies are presumably for the surgery to carry out consultancy services, not for individual GPs to act as consultants? Wouldn’t the latter be reported under individual payments? | This is correct. We have now clarified the sentence in question (p.18, para 3). |
| 13 | Table 1: Given the large skew in the distribution of payment numbers to surgeries, I wondered whether some of this data might be better graphed than presented within a table according to quartiles. I missed being able to know actual numbers of surgeries (or proportion of surgeries with the total n provided) with different numbers of payments and/or value of payments. It would also be good to group receipt of payment by surgeries in one section (or figure) and payments by companies in another as each denominator is quite different. Denominators are also needed for numbers of surgeries and numbers of companies and it would be good to have information on the total numbers and total values of payments within the table. | Thank you for your suggestion, we have considered visualising Table 1 by creating 5 histograms of the distribution of individual payments, payments per GP practice, payments per companies, practices per companies, and companies per practice. However, due to the extreme skewness of the payment values and numbers, the histograms were not informative and we decided to keep the table instead but have made some changes to make it clearer.  The proportion of surgeries and companies are indicated by the IQR. For example, the table shows that 25% of practices receive £217.25 or less and that 25% receive 1 payment only. |
| 14 | I also missed information on how this subset of surgeries relates to all surgeries in the UK. What proportion have received payments? Is there a regional skew in terms of proportions of surgeries receiving payments? | Information on the proportion of practices receiving payments in the different regions has been added to Table 3 and differences between the regions in terms of the share of practices receiving payments has been explained in the text (p. 13, para 2). |
| 15 | P12: The following sentence is unclear: “Of the 10 companies, Bayer was clearly dominant both in relation to the number (43.41% of the top 10) and value of payments (31.96% of the top 10) and the number of surgeries to which payments were made (44.63% of the top 10).” What does ’43.41% of the top 10’ refer to exactly? Perhaps instead just say that Table 2 describes the top 10 companies by payment amount and that Bayer provided the largest amount. | In that sentence we wanted to show that Bayer stood out not only from all companies but also from the top 10 donors. Since it might be hard to follow for the reader, I deleted those numbers from the sentence (p 12., para 1). |
| 16 | Table 2 should be simplified. I would suggest leaving off most of the columns on quartiles etc. and just retaining the first 3 column on total amount paid, number of surgeries and number of payments. You could list the payment value median [IQ range] as a fourth column. Headings could be clearer and I did not understand what the percentages referred to – clearly not a percentage of total value of payments to GP surgeries. Additionally when you say ‘all HCO’ do you mean all surgeries only or all types of health care organisations? I would suggest just focusing on the surgeries in this table. | Table 2 has been updated. |
| 17 | When you refer to most payments being under 869 pounds, I’d suggest instead stating the proportion under 1000 pounds or another more standard number. | It is the third quartile, that is why we are saying that most payments are under £869. We refer to numbers and values in terms of quartiles throughout the text, therefore we think we should keep the description accordingly (p. 12, para 2). |
| 18 | Page 14 describes the proportion of surgeries receiving payments in different regions. It would have been helpful to state at the beginning of the results section what proportion of surgeries the 1790 represents. My recommendation would be to shift this to the beginning of the results section and to include the proportion per region in your table, with total numbers of surgeries per region also stated. Table 3 could easily include this information in the middle column.  An alternative would be to use the table you now call S1 in the text rather than Table 3. It provides key information – text in headings could be cut down. | In the beginning of the results section we explain that the original Disclusre UK dataset includes payments to 1,790 practices (p. 10, para 3). After exclusion (the reason and process of which is also explained in p. 7, para 2) we conducted analysis on 1,643 practices. How these 1,643 practices are divided in the different regions are show in Table 3. The proportion of these practices receiving payment out of the total practices in the different regions has now been added to Table 3.  We would like to keep this table where it is because first we explain the overall distribution of payments across practices and companies and then we move on to explaining differences based on practice characteristics (region, MDI, patient demography). |
| 19 | Table 3: For the three sections on quartiles at the bottom, rather than saying ‘first quartile’ to 4th quartile’ you could use for example ‘Socio-economic index’ as a header row and then say ‘lowest deprivation [1st quartile] to ‘highest deprivation’ and similar for patients over 65. If these are quartiles you do not need to say numbers of surgeries per quartile. A header row ‘region’ at the top would also mean you could organize your columns differently. No need to put ‘breakdown of’ in header rows. | All changes have been made to Table 3. |
|  | Page 15 – SNA is used without being defined. | SNA stands for social network analysis. The abbreviation, as well as the description of this method is explained in the introduction(p. 5, para 2). The subtitle of that Results section is called “Social network analysis of connections between drug companies”, therefore we believe the abbreviation does not need to be defined again. |
| 20 | Network analysis: This entire section needs a rethink in terms of what the value to the reader of this network analysis is. I find the concepts of centralisation and density difficult to interpret in terms of the added knowledge on patterns of industry payments to GP surgeries. I can see that this is a way to measure the extent to which surgeries receive funding from many different companies and whether companies tend to have similar patterns of funding but beyond this the density and centralisation levels do not add meaning.  The description and meaning needs to be clearer. What does the high centrality score add for Bayer as compared with the data you have already presented on this company having the highest value of payments?  Additionally, the discussion of competing companies may be overinterpretation, as a company with products treating the same condition would be directly competing; others might just be targeting the same surgeries. | The network analysis results section has been almost completely rewritten. The aim of the SNA is now to show the shared interest of companies in the same practices and their strategies in building presence in these practices, by focusing on the value of payments only. The concepts of centrality, density and centralisation have been explained better with the help of the network graphs, which were also added replacing Table 4. |
| 21 | There appear to be different patterns by company of making many small payments versus fewer large payments. This could be a vestige of companies’ reporting patterns. Some may be more likely to break down their reports of payments to surgeries into many smaller subsets; others to amalgamate. In order to understand whether these patterns of payment size are meaningful, you would need to check the Disclosure UK reporting rules to see whether the extent of flexibility.  Our analyses of companies' payments in Australia to patient organisation suggested large differences in reporting patterns per company that were not necessarily reflective of actual differences (e.g. some seemed to amalgamate more than others). This suggested to us that value of payments per organisation was much more reliable as a measure than numbers of payments. I don't know the UK data, but if this is the case in the UK as well it should guide your analysis and interpretation. | As we explain in the discussion (p. 21 para 1), the UK is the only European country in which the pharmaceutical industry trade group requires its members to report itemised payments to heathcare organisation. This requirement was included in the ABPI Code from 2015, which coincided with the time when the version of the Disclosure UK we analysed was compiled. The ABPI does not provide – to the best of our knowledge – detailed guidance on payment itemisation but we have no reasons to believe that there are significant differences between companies in terms of how they itemise payments |
| 22 | P18: exploration of company payments and drug marketing. I’d suggest leaving this section out as it is not directly relevant to the focus of this paper and it is a stretch to assume that these specific payments are related to the drugs with promotion that has been found to breach the industry code. A reference to the Bayer breaches for this anticoagulant (please use the generic name) might be brought in within the discussion instead, when commenting on Bayer being the company with the highest value payments to GP surgeries | Thank you for this important comment. Please see our response to point 6 which also addresses this comment. |
| 23 | Discussion The beginning of the discussion includes information that should be in the results. The fact that GP surgeries are the 5th type of organisation by amount of funding is important. It also refers to a table (Table S2) which states total amount of funding to GP surgeries – this funding amount also should be in the text. | The rank of surgeries has been added to the results (p. 10, para 3) and the total value of payments made to practices has been added to the discussion (p. 18, para 2). |
| 24 | Table S2 is much too long and detailed. You could stick to the top 10 organisations. Please shorten some names e.g. ‘multi-profession associations’ (4th). | Table S2 has been corrected (now S3). |
| 25 | P24: As noted above, the network analysis may not reflect direct drug company competition. More information is needed about which drugs are being promoted, to treat which conditions, per company, before that statement can be made. It is also possible that companies are promoting to the same surgeries for different treatment areas. | The word “competition” has been deleted from the text. The focus has been turned to “shared interest” of companies and interest in “building presence” in practices. |
| 26 | Table S3 could be simplified (e.g. only include full data rather than having two rows with full and after exclusions), but do state the denominator (# surgeries) for the table. It is useful to know the breakdown of amounts per type. | Table S3 has been corrected (now S4). |
| 27 | A general note: there are a number of typos in the text which need to be corrected. Secondly, most tables that include all of the quartiles plus minimum and maximum value and median could be simplified into a single column. This could be ' median [interquartile range]' or 'median [min- max]'. . An example is S4 – but this applies across the board to tables within the text and the supplement. A note for S4 – do not include the practice code unless this would be meaningful to readers. | Typos have been corrected.  IQR has been added to all tables instead of Q1 and Q3.  Practice codes have been deleted. |

|  | **Reviewer 2’s comments** | **Authors’ response** |
| --- | --- | --- |
| 1 | The biggest suggestion I have is that the piece could be reduced in length, and at the same time made clearer. Related to this, the Methods needed to be disentangled from the Introduction and the Results need to be disentangled from the Methods.  A statement on whether or not there was a pre-existing protocol for the study, is needed.  And an important caveat is that I have no biostats specialty, so I have not reviewed the stats. | Thank you for all your helpful comments. We have sought to reduce the wordcount wherever possible, including by deleting the section on the PMCPA investigations into Bayer cases from the findings.We have also streamlined the discussion.  We have also sought to disentanglethe different sections.  The statement about writing a protocol about the data extraction process has been added to the methods section (p. 8, para 1). |
| 2 | Page 4 (P4), Paragraph 1 (Pa1) You might consider referencing the recent material in BMJ, which summarises all the evidence about the distorting impacts of COIs, and flags the BMJ campaign to push for more independence. ( <https://www.bmj.com/content/367/bmj.l6576> ) | Thank you for this important recommendation. The article has been added to the Policy recommendations section. |
| 3 | P6 – First main paragraph: this feels like material that should best be in Methods.  P6 Last sentence. This sentence seems to be preempting results – as presumably you discovered this information during study – so again – feels more like methods. | At the end of the introduction, we summarise our assumptions based on the relevant literature and in the methods section we describe how we want to test those assumptions by analysing the data. Moving the assumptions from the introduction would make the text more confusing. |
| 4 | P7 Methods. I would start with an overall description of the study design- before going into specifics. Also, it is important to mention whether or not there was a pre-specified protocol for the study – and if so then later describe any deviations from it. | A “Study design” section has been added to Methods outlining the overall study design. We did not write a protocol for the study design and therefore we could not deviate from an original plan. We made a detailed description of the steps of data cleaning and the combination of different datasets. |
| 5 | P8, 1st paragraph. Some of this material seems to be best in results section – forgive me if I am confused, btu it feels like results are being given (in terms of numbers of pounds etc) in the middle of the Methods section. Eg this sentence “Of the total of 1,791 distinct surgeries, receiving 2,944 payments, worth £2,730,261.32, 147 (8.95%) surgeries, receiving 197 (6.69%) payments, worth £170,595.29 (6.24%), could not be linked to a practice code from the FFT dataset” | The reason we state the number and value of total payment and excluded payments is to help the reader understand how much of the data was excluded from the analysis. We are not talking about the excluded data in the results, therefore we would prefer not deleting this information from the methods section. |
| 6 | Table 2. Typo in title of Column 2: “Nnumber”  P14 typo “patters” | This has been corrected, thank you. |
| 7 | P14 Forgive me if I missed something – but this statement on deprived areas seems to contradict the statement below from the abstract? Unless I have misunderstood? “Contrary to our expectation that surgeries in more deprived areas will obtain more payments due to the greater number of prescriptions per patients in these regions, our data shows that surgeries in the most deprived areas (1st quartile based on MDI) received the smallest, while surgeries in the top two quartiles - the largest amount of payments”  From Abstract: “Surgeries with more patients, a greater proportion of elderly patients, and those in more deprived areas received more payments on average” | The mistake in the abstract has been corrected. Practices in more deprived areas received less payments. |
| 8 | Table 3. Is there something missing from this line? “4 th quartile 411 434.50 – 2283.12)” | We corrected this mistake. |
| 9 | P19 – I would consider removing the detailed list of the 4 breaches, and try and summarise briefly the nature and importance of the breaches and the issues/dangers associated with the drug and/or its promotion. (I note there is relevant material in Discussion, but I would still summarise rather than list the 4) | Thank you for this comment. In light of this comment and comments from the other reviewer, we have removed the analysis of PMCPA cases from the analysis. We now only mention them in the discussion as a way to hypothesise about the products behind Bayer’s payments and to illustrate problems of unethical marketing in the UK |
| 10 | P20 – Typo “payment patter” (unless “patter” has a meaning I am unaware of)  P24 Typo “payments” | This has been corrected |
| 11 | P26. As mentioned earlier, there seems an opportunity here to be calling for more independence. It is in a way unbelievable that the NHS still allows companies to make payments to people making decisions about those companies’ drugs. The BMJ campaign could be considered, but that is up tto the authors. ( <https://www.bmj.com/content/367/bmj.l6576> | The article has been added to the Policy recommendations section. |
