# Supplementary material for: Drug company payments to General Practices in England: Cross-sectional and social network analysis
Source: PLoS One. 2021 Dec 7;16(12):e0261077. doi: 10.1371/journal.pone.0261077 (PMC8651134; doi:10.1371/journal.pone.0261077)
Supplement: Attachment — Submitted filename: Responding to comments_20.11_ES.docx [file pone.0261077.s011.docx]

|  | EDITOR’S COMMENTS | AUTHORS’ RESPONSES |
| --- | --- | --- |
| 1 | Page 3, 7^th^ line from bottom: Delete “a” after “variety”. | Thank you! This has been corrected. |
| 2 | Page 4, 7^th^ line from top: Explain the term "healthcare commissioners". | Thank you for helping us clarify this point. We have replaced the term "healthcare commissioners" with “organisations commissioning (or procuring) healthcare services for patients” (p. 4, first para). |
| 3 | Page 4, 9^th^ line from bottom: Does the term “practices” mean exclusively GP practices or does it also include specialists' practices? | We have clarified this by stating that this term means exclusively GP practices (p. 4, first para). Thank you for noting this point as with the increasing trend towards the privatisation of healthcare services in England, non-GP consultant-led services may be gaining prominence in the future. Therefore, it makes sense to ensure our focus on GP practices is as clear as possible. |
| 4 | Page 6, last paragraph before Methods: Considering that you devote 3.5 pages in the Results to your network analysis you need to make a stronger case here about why this type of analysis is important. | We have reformulated the opening sentence in the paragraph focusing on SNA and also added a further sentence to justify the need for its use (p. 5, second para). |
| 5 | Page 9, first line: It should be “calculated”. | Thank you! This has been corrected. |
| 6 | Page 10, second line: I would suggest moving the sentence beginning "More frequent payments..." and the next paragraph to the end of the Introduction to help make the case why network analysis is important. | Thank you for this helpful recommendation. We have now integrated our interpretation of the number and value of payments with the paragraph explaining the importance of SNA as one of our approaches to analysis in the Introduction (p. 5, second para). |
| 7 | Page 10, first line in Results: In the Methods you've already pointed out that your analysis is not based on 1790 practices and you've given the reason. I'd suggest eliminating this sentence and then moving up the first sentence in the next paragraph to start the Results section. | Thank you! This has been corrected. |
| 8 | Page 11, 4^th^ line: Insert “in total” after “payments”. | Thank you! This has been corrected. |
| 9 | Page 15, 6^th^ line: Is there any way of determining if companies with a shared interest are comarketing certain drugs? | Thank you for this question. This is a very interesting matter, which could help us interpret our findings more deeply. However, unfortunately, we are not aware of any way to assess the co-marketing of drugs in the UK. |
| 10 | Page 18, 3^rd^ line from bottom: It should be “A similar trend…” | Thank you! This has been corrected. |
| 11 | Page 19, 11^th^ line from top: It's unclear what is meant by "recipients' peers". | Thank you! The term has been explained in the text. |
| 12 | Page 19, 6^th^ line from bottom: “Medicines” is misspelled. | Thank you! This has been corrected. |
| 13 | Page 19, 2^nd^ line from bottom: It should be “covers”. | Thank you! This has been corrected. |
| 14 | Page 21, 7^th^ line from bottom: Insert “to” between “payments” and “HCOs”. | Thank you! This has been corrected. |
| 15 | Pages 22-23: I think that there is too much space devoted to the issue of unethical marketing of DOACs in general and rivaroxaban in particular, especially given the unknown relationship between rivaroxaban marketing and payments to GP practices. | Thank you! We have significantly shortened the section. This is also helps us shorten the text (beginning on p. 20, last para). |
| 16 | Page 25, 11^th^ line from bottom: Insert “a” after “Such”. | Thank you! This has been corrected. |
| 17 | Page 25, 7^th^ and 6^th^ lines from bottom: Would such a central register make a difference to patients' or doctors' behaviour? Research in the US suggests that it doesn't. | Thank you. We have introduced this very important point towards the end of 1^st^ paragraph on p 24. |
| 18 | Page 25, 6^th^ line from bottom: Insert “for” between “particular” and “medicines”. | Thank you! This has been corrected. |

|  | Reviewer 1’s comments | Authors’ responses |
| --- | --- | --- |
| 1 | Abstract: The reference to the 'top 10 donors' does not flow that well from previous text, and the reader needs to consider this has switched from being about practices to being about companies. Perhaps instead (addition in caps): Payments to practices were highly concentrated, AND SPECIFIC COMPANIES WERE ALSO HIGHLY DOMINANT. The top 10 donors and the top 10 recipients amassed 87.9% and 13.6% of the value of payments, respectively... | Thank you for this useful suggestion. We have implemented it. |
| 2 | Table 1: 'value of individual payments' could be misread as being about payments to individuals. Perhaps 'value of individual payments to each practice' or 'single payment value' | Thank you. This has been corrected. |
| 3 | Also please left justify the row labels, similarly to table 2. | We appreciate this kind suggestion. We have now changed the table formatting as suggested. |
| 4 | page 17: this line is unclear: "This suggests that there is an overall lower interest in the same practices as expressed by higher value payments." Do you mean that if practices received higher value payments from one company, they were less likely to also receive higher value payments from other companies? Please clarify. | Thank you for noticing this. It means that one practice does not receive high value payments from multiple companies. We have now added this to the text (p.16, first para). |
| 5 | Page 18: This line assumes too much reader recall of newly defined technical terms, especially given the similarity of terms: "Interesting differences can be observed in terms of centralisation and centrality," Interesting differences can be observed in terms of centralisation (BRIEFLY REDEFINE) and centrality (BRIEFLY REDEFINE), | Thank you for helping us guide the readers more effectively. We have added these brief definitions as suggested (p.17, second para). |

|  | Reviewer 2’s comments | Authors’ responses |
| --- | --- | --- |
| 1 | My first main comment in my initial review was that the original paper was too long and could be reduced in length. The authors have responded that they have “shortened wherever possible”. I do not agree, and I think the piece is still too long. The revised Introduction alone seems to be still close to 1000 words long. The Methods section also feels too long in places, such as the data sources and social network analysis sections. It is an important study, but in my view does not warrant an exceptionally lengthy write-up. | We have indeed shortened our paper whenever possible. For example, as already explained in our previous responses, we removed an entire section from the findings focusing on Bayer’s marketing and moved its shorter version to the Discussion. It is also important to note that additional material has been added in response to some of the comments made by the Editors and the other Reviewer, which has made any word count reduction less apparent. For example, we have been asked to repeat information regarding some aspects of SNA as readers are likely to be unfamiliar with this methodology.  To respond to your specific concerns, in the current version of the manuscript the Introduction has 958 words but this results from expanding the justification for the use of SNA and moving some material from the Methods to the Introduction, as suggested by the Editors. The Methods, however, have been shortened from 1034 words to 911 words and the Discussion from 2004 words to 1572 words (again, despite adding some new material). The Findings section increased slightly from 1392 to 1470 words, due to additional clarifications and definitions asked by the reviewers. We hope that this overview demonstrates our commitment to shortening the article, while bearing in mind that PLOS One does not have a fixed word count limit. |
| 2 | My second main comment is that the revision should be clearer. I feel a further revision could make the whole manuscript clearer still. | Although we were not entirely sure what exactly you meant by “clarity” in this instance, we have sought to maximise clarity while shortening the manuscript in current round of revisions. |
| 3 | My third main comment was a suggestion to try and disentangle different sections, and remove overlap between Introduction, Methods and Results. The authors responded in their letter accompanying their revision that “We have also sought to disentangle the different sections.” Yet, if I am not mistaken, in two of the three specific suggestions which I made to disentangle, they have responded that this is not possible. I think authors need to revisit my suggestions in their next revision, particularly in relation to the payment figures currently included in the Methods. | In the previous version of the manuscript, we did include some figures in the methods as we used them to support our points about data management and integration, and as such reported them as part of the methods. We have now, however, moved these figures to the opening part of the methods, following your suggestion (p. 10, first two paragraphs).  While we appreciate that you may have a different perspective on what might belong to either of the sections, we cannot see any further potential “overlaps” or “entanglement” between the Introduction and Methods or between the Methods and Results. |
| 4 | My fourth main comment, and a very important one, pertained to the issue of a pre-existing protocol. I wrote in my initial review “A statement on whether or not there was a pre-existing protocol for the study, is needed.” The authors responded to my comment by writing: “The statement about writing a protocol about the data extraction process has been added to the methods section (p. 8, para 1).” And the new revised manuscript stated “The data extraction process followed a detailed protocol which can be obtained from the authors upon request.” I am concerned because I did not suggest making a statement about a protocol for “data extraction”, I suggested making a statement about a pre-existing protocol for the study. I think any revised manuscript needs to be crystal clear about whether there was a pre-existing protocol for the study, and potentially offer an explanation for why there was not one. Moreover, if there was a pre-existing protocol for part of the study, ie the data extraction, potentially this could be made available as a supplementary file. | We apologise for misunderstanding your fourth comment. There was no pre-existing protocol for the study, which we now make absolutely clear at the beginning of the Methods section (p. 6, last para). Our research was exploratory, and we did not know a priori which publicly available datasets with general practice characteristics could be matched with the drug company payments and how.  As suggested, we have now included the protocol for data extraction as a supplementary file (Appendix 2) – mentioned on p. 8, first para. |
